# Osteoclast-derived apoptotic bodies couple bone resorption and formation in bone remodeling

**DOI:** 10.1038/s41413-020-00121-1

**Published:** 2021-01-11

**Authors:** Qinyu Ma, Mengmeng Liang, Yutong Wu, Fei Luo, Zaisong Ma, Shiwu Dong, Jianzhong Xu, Ce Dou

**Affiliations:** 1 Department of Orthopedics, Southwest Hospital, Third Military Medical University, Chongqing, 400038 China; 2 Department of Biomedical Materials Science, Third Military Medical University, Chongqing, 400038 China; 3 Department of Orthopedics, General Hospital of Xinjiang Military Command, Urumqi, Xinjiang 830000 China; 4 State Key Laboratory of Trauma, Burns and Combined Injury, Third Military Medical University, Chongqing, 400038 China; 5 Department of Orthopedic Surgery, Johns Hopkins University School of Medicine, Baltimore, MD 21205 USA

**Keywords:** Bone, Homeostasis

## Abstract

Bone remodeling is precisely coordinated by bone resorption and formation. Apoptotic osteoclasts generate large amounts of apoptotic bodies (ABs) marking the end of the bone resorption phase, whereas the functions of osteoclast-derived ABs remain largely unknown. Here, we identified the molecular profile of ABs derived from osteoclasts at distinct differentiation stages and investigated their corresponding functions. ABs were isolated from apoptotic bone marrow macrophages, preosteoclasts, and mature osteoclasts induced by staurosporine. Proteomic signature analysis with liquid chromatography-tandem mass spectrometry suggested marked protein cargo differences among the different ABs. Further bioinformatic analysis showed that the proteomic signatures of the ABs were highly similar to those of their parental cells. Functionally, pOC-ABs induced endothelial progenitor cell differentiation and increased CD31^hi^Emcn^hi^ endothelial cell formation in a murine bone defect model via their PDGF-BB cargo. mOC-ABs induced osteogenic differentiation of mesenchymal stem cells and facilitated osteogenesis via RANKL reverse signaling. In summary, we mapped the detailed proteomic landscapes of ABs derived from osteoclasts and showed that their potential biological roles are important in coupling bone formation with resorption during bone remodeling.

## Introduction

Billions of cells undergo apoptosis every day to maintain physiological homeostasis in the human body^1^. At the late stage of apoptosis, the nucleus and cytoplasm of apoptotic cells are compacted and disassemble into subcellular membrane-bound extracellular vesicles (EVs) named apoptotic bodies (ABs)^2,3^. As a subset of EVs, ABs (1–5 μm) are much larger than exosomes (100–200 nm) or microvesicles (MVs) (100–1 000 nm) and are generated only by apoptotic cells^4^. The assembly of organelles such as the endoplasmic reticulum, nuclear contents, and mitochondria into ABs is considered a random process^5,6^; therefore, ABs may contain various materials such as proteins, lipids, and RNAs^7,8^. Several autoantigens, such as complement proteins (C1QC and C3B) and histone family members (especially histone 2B and histone 3), have been found to be highly enriched in ABs and are therefore commonly considered markers to differentiate ABs from other subtypes of EVs^9,10^. Traditional perspectives view ABs as a group of garbage bags that encapsulate remnant fragments of dead cells and are subsequently phagocytized to prevent adverse impacts on their environment. However, accumulating evidence supports the participation of ABs in biological events, including inflammation, autoimmunity, and cancer, by regulating recipient cells^11,12^. These findings demonstrated that ABs not only are cell debris but also are involved in intercellular crosstalk. However, studies revealing the precise composition of AB cargo are lacking, and the detailed mechanisms underlying the biological roles of ABs remain unknown.

Osteoclasts are bone-resorbing cells that play important roles in bone remodeling and metabolism^13^. Activated by two critical factors, namely, receptor activator of nuclear factor κB ligand (RANKL) and macrophage colony-stimulating factor (M-CSF), macrophage progenitor cells differentiate into mononuclear preosteoclasts (pOCs), and multiple pOCs further fuse to differentiate into multinucleated mature osteoclasts (mOCs)^14^. Although both are called osteoclasts, the functions of pOCs and mOCs are quite different. pOCs generally do not show a significant bone resorption function but can secrete anabolic cytokines, which mediate intercellular crosstalk with endothelial progenitor cells (EPCs) to promote angiogenesis by releasing PDGF-BB^15^. On the other hand, mOCs, in addition to their bone resorption activity, show strong potency in promoting osteogenesis through RANKL reverse signaling^16,17^. Our previous studies further showed that pOCs and mOCs have distinct transcriptome and small RNA signatures^18^. In vivo, osteoclasts have a relatively short lifespan of only a few weeks before apoptosis^19,20^. During bone remodeling, osteoclasts undergo apoptosis at the end of the bone resorption phase and produce large amounts of ABs; this event is followed by the movement of osteoblasts into the resorption space, indicating the initiation of the bone formation stage^21,22^. Previous findings have centered around the process in which osteoclasts couple with osteoblasts by releasing cytokines, secretory proteins, and exosomes^23–25^. However, the effects of osteoclast-derived ABs on their environment and cell coupling remain unclear.

Here, we mapped the whole-proteome signatures in osteoclast-derived ABs. Through bioinformatic analysis, we showed marked protein cargo differences among different ABs and revealed the corresponding proteomic signatures. Comparison of the whole proteome of these cells with that of the respective parental cells revealed high signature similarity. This similarity was further confirmed at the functional level via in vitro and in vivo experiments.

## Results

### Isolation and characterization of ABs from bone marrow macrophages (BMMs), pOCs, and mOCs

**Fig. 1:**
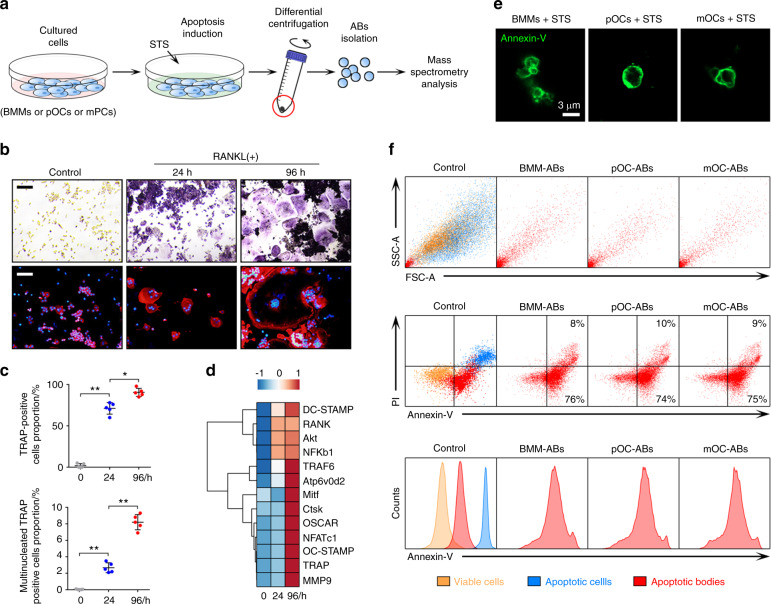
Isolation and characterization of ABs from BMMs, pOCs, and mOCs. **a** Graphical illustration of the procedure for AB isolation. **b** Images of TRAP and IF staining of the cytoskeleton of BMMs, pOCs, and mOCs. Cell nuclei stained with DAPI are shown in blue, and vinculin is shown in red. The scale bars represent 200 μm. **c** The proportions of TRAP-positive cells and multinucleated TRAP-positive cells were quantified in each well (96-well plate), *n* = 5. **d** Heat map showing the expression profile of RANKL-dependent specific osteoclastogenic genes from 0 h (BMMs) to 96 h (mOCs). **e** Representative images of ABs stained with Annexin-V/FITC. The bar represents 3 μm. **f** FSC/SSC analysis and Annexin-V/PI analysis of viable cells, apoptotic cells, and ABs. The data in the figures are presented as the average ± SD values. Statistically significant differences between the treatment and control groups are indicated as * (*P* < 0.05) or ** (*P* < 0.01)

We isolated ABs derived from osteoclasts at different stages according to the outlined experimental design (Fig. 1a). Whole bone marrow cells (BMCs) obtained from the hind limbs of 11-week-old male mice were stimulated with M-CSF for 48 h to generate BMMs. BMMs were then induced to differentiate into pOCs (24 h after RANKL and M-CSF stimulation) and mOCs (96 h after RANKL and M-CSF stimulation), as validated by tartrate-resistant acid phosphatase (TRAP) staining and immunofluorescence (IF) staining of the cytoskeleton (Fig. 1b). Quantitative analysis showed that TRAP-positive cells accounted for more than 80% of the total cells at 24 h and for almost 100% at 96 h, while actin ring-positive multinucleated cells (cells with more than three nuclei) accounted for less than 3% at 24 h but increased to 8% at 96 h (Fig. 1c). Gene array analysis showed marked increases in the expression levels of most RANKL-dependent genes in pOCs and mOCs, confirming the distinct stages (Fig. 1d). ABs from BMMs, pOCs, and mOCs were isolated after apoptosis was induced by staurosporine (STS)^26^. Cells undergoing apoptosis were characterized by reduced cytoplasmic refraction and obvious membrane blebbing (Fig. S1). After apoptosis induction, differential centrifugation was used to separate AB-sized EVs from dead cells, cell debris, and other small EVs^27^. For AB identification, we stained ABs with Annexin-V/FITC and observed them using confocal microscopy. ABs were thus characterized as Annexin-V-positive spherical vesicles with monolayer membranes (Fig. 1e). We next used flow cytometry to determine the purity of the isolated ABs (Fig. 1f). Forward/side scatter (FSC/SSC) analysis showed a significant size difference between ABs and cells. Annexin-V/FITC analysis showed that relative to apoptotic cells, ABs expressed intermediate levels of phosphatidylserine (PS), whereas viable cells showed almost no exposed PS. In addition, propidium iodide (PI) staining identified a small PI-positive AB subset, implying that a small fraction of—but not all—ABs contain nuclear DNA. No significant differences were observed in size or Annexin-V intensity among different ABs.

### Proteomic profiling of ABs derived from BMMs, pOCs, and mOCs

**Fig. 2:**
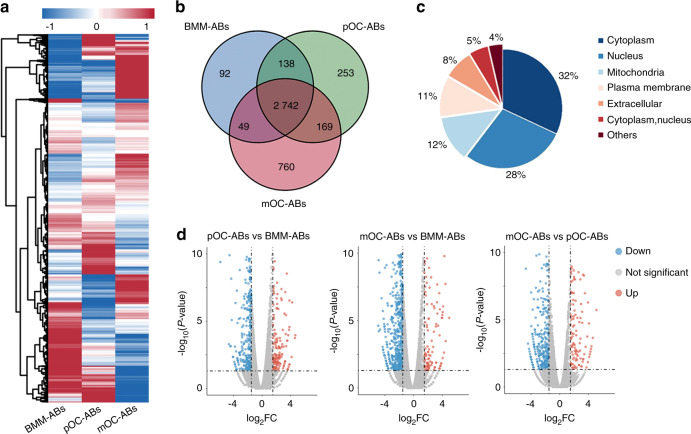
Proteomic profiling of ABs from BMMs, pOCs, and mOCs. **a** Heat map visualization of hierarchical clustering showing the expression profile of 4 306 proteins identified in ABs. **b** Venn diagrams comparing the total proteomes of different types of ABs. **c** Subcellular localization analysis showing the intracellular distribution of all DEPs. The DEPs were localized mainly in the cytoplasm, nucleus, and mitochondria. **d** Volcano plots showing the DEPs in the three comparison groups. The red dots represent DUPs (FC > 2.0), and the blue dots represent DDPs (FC < 0.5)

To establish the proteomic landscape of osteoclast-derived ABs, AB samples were lysed and digested for liquid chromatography-tandem mass spectrometry (LC-MS/MS) analysis. Raw data were processed using MaxQuant software with the database set to Mouse_Swissprot_1808^28^. In total, 25 623 peptides and 4 306 proteins were detected, and the corresponding proteomic profiles of the three AB types after hierarchical cluster analysis are presented as heatmaps (Fig. 2a). Among the detected proteins, 3 020, 3 301, and 3 720 were identified in ABs derived from BMMs, pOCs, and mOCs, respectively. Venn diagram analysis further revealed both intersected and distinct protein signatures in different ABs (Fig. 2b). Three comparison groups were established: pOC-ABs *vs* BMM-ABs, mOC-ABs vs BMM-ABs, and mOC-ABs *vs* pOC-ABs. All differentially expressed proteins (DEPs) were statistically significant (*P* < 0.05), with fold changes (FC) of greater than 2.0. The distribution of DEPs in the three comparison groups is shown in Fig. S2a. These DEPs were associated mainly with cellular components, including the cytoplasm, nucleus, and mitochondria (Fig. 2c). All DEPs among the three groups were identified and visualized in volcano plots (Fig. 2d).

To better understand the function of AB protein cargo, we further divided the DEPs into differentially upregulated proteins (DUPs, FC > 2.0) and differentially downregulated proteins (DDPs, FC < 0.5). Regarding DUPs in pOC-ABs and mOC-ABs relative to BMM-ABs, the number of DUPs associated with the cytoplasm in pOC-ABs (33.8%) was almost equal to that associated with the nucleus (31.2%), while the number of DUPs associated with the cytoplasm in mOC-ABs (44.3%) was significantly higher than that associated with the nucleus (20.5%) (Fig. S2b, d). Regarding DUPs in mOC-ABs relative to pOC-ABs, the number of DUPs associated with mitochondria in mOC-ABs (22.8%) was significantly increased and was even higher than the number of DUPs associated with the nucleus (21.1%) (Fig. S2e, f). Regarding DDPs in pOC-ABs and mOC-ABs relative to BMM-ABs, the numbers of DDPs associated with the nucleus in both pOC-ABs (38.7%) and mOC-ABs (41.1%) were significantly higher than those of DDPs associated with the cytoplasm (24.2% and 24.9%, respectively) (Fig. S2c, e). These results suggested a significant difference in the subcellular localization of DEPs among the three ABs. In brief, BMM-ABs had a higher abundance of nuclear proteins, whereas pOC-ABs contained more cytoplasmic proteins, and mOC-ABs were rich in mitochondrial proteins. Gene ontology (GO) enrichment analysis showed the predicted functions of the DEPs in these ABs. The GO terms were categorized into biological process (BP), cellular component (CC), and molecular function terms and ranked by enrichment factor [-log_10_*(P* value)]. DUPs in pOC-ABs relative to BMM-ABs were enriched in BP terms correlated with cell differentiation and development, and DDPs in pOC-ABs were enriched in the term cell response to external stimulus (Fig. S3a, b). In contrast, DDPs in mOC-ABs relative to BMM-ABs were enriched mainly in immune system development and the immune response (Fig. S3). DUPs in mOC-ABs relative to pOC-ABs were enriched mainly in cell metabolism, while DDPs in mOC-ABs relative to pOC-ABs were associated mainly with cellular transport (Fig. S3e, f). Basically, these differences may be caused by the different functions of the parental cells, as pOCs are still actively in differentiating during osteoclastogenesis, whereas compared with monocytes/macrophages, mOCs have lost most of their immunological characteristics and properties^29^. These results allowed us to construct a proteomic map of these three types of ABs and suggested distinct biological functions of different ABs.

### Similarities between the proteomic signatures of ABs with those of their parental cells indicate functional similarities

**Fig. 3:**
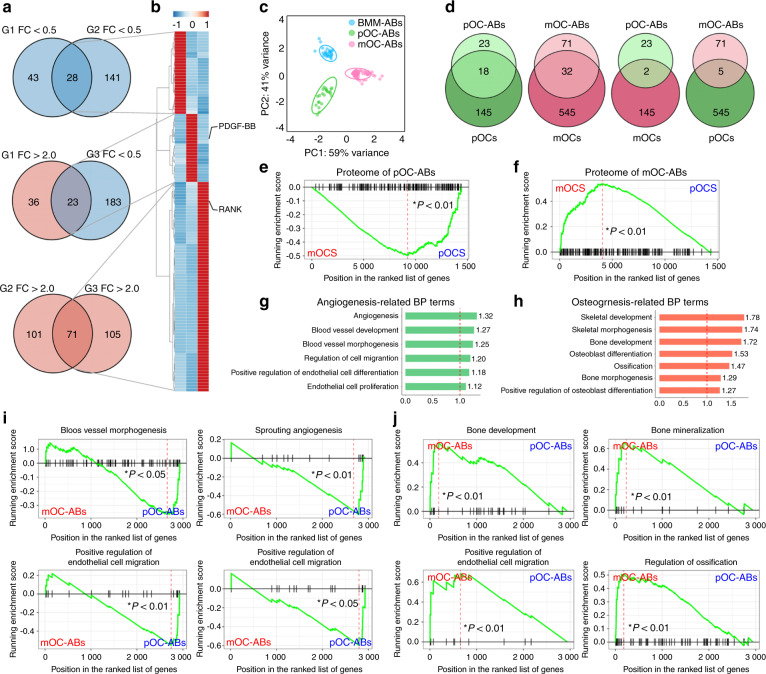
The similarities of the proteomic signatures of ABs with those of their parental cells indicate functional similarities. **a** Venn diagram analysis of the three comparison groups identified the proteomic signatures of the different types of ABs. The threshold FC was >2.0 or <0.5. (G1, pOC-ABs *versus* BMM-ABs; G2, mOC-ABs *versus* BMM-ABs; G3, mOC-ABs *versus* pOC-ABs). **b** The proteomic signatures of ABs derived from BMMs, pOCs, and mOCs are shown as a heat map. Among the proteins, PDGF-BB and RANK were signature proteins of pOC-ABs and mOC-ABs, respectively. **c** PCA of the protein expression signatures of the three types of ABs. The elliptical solid lines indicate the 95% confidence intervals. **d** Venn diagrams comparing the candidate protein signatures of ABs with the gene signatures of their corresponding parental and nonparental cells. GSEA plots showed the relationship of the whole-proteome signatures of (**e**) pOC-ABs and (**f**) mOC-ABs with the gene expression profiles in the corresponding parental cells. GO analysis revealed that (**g**) DUPs in pOC-ABs were enriched in angiogenesis-associated BP terms, whereas (**h**) DUPs in mOC-ABs were predominantly enriched in osteogenesis-associated BP terms. GSEA showed that (**i**) enriched proteins in pOC-ABs were more closely associated with angiogenesis-related biological processes, whereas (**j**) enriched proteins in mOC-ABs were associated with osteogenic activities

To further clarify the specific functions of different ABs, we performed Venn diagram analysis of DEPs to determine the specific AB proteomic signatures (Fig. 3a). In this way, we identified 28, 23, and 71 specific signatures in ABs derived from BMMs, pOCs, and mOCs, respectively. These signatures were subsequently presented as heatmaps after hierarchical cluster analysis (Fig. 3b). Principal component analysis (PCA) was performed to detect the signature-to-signature distances, revealing significant separation of clustering among the signatures of these different types of ABs (Fig. 3c). GO enrichment and subcellular structure localization analysis of the AB protein signatures further revealed that the differences were in both functions and components (Fig. S4). We then used our previously reported gene expression data from BMMs, pOCs, and mOCs to identify the relationship between ABs and their corresponding parental cells^18^. Intriguingly, of the 122 identified AB signatures, most of the candidate genes were also found to be highly expressed in the corresponding parental cells. Venn diagram analysis showed that the AB protein signatures exhibited more significant overlap with those of the corresponding parental cells (5.5%–9.6%) than with those of nonparental cells (0.8%–1.1%) (Fig. 3d). Then, we performed gene set enrichment analysis (GSEA) to determine whether the unions of these protein signatures reflect parental cell-like profiles and found that the signatures of both pOC-ABs and mOC-ABs exhibited high and specific similarities relative to those of the parental cells (Fig. 3e, f). To determine whether the proteomic signature and profile similarities between ABs and their parental cells results in functional similarities, we performed GO analysis. The most enriched GO terms in pOC-ABs were associated with angiogenic activities, while those in mOC-ABs were osteogenesis terms (Fig. 3g, h). We then assessed AB functions by GSEA on selected predefined gene sets of BP known to be specific to the parental cells. Intriguingly, the GSEA results showed that pOC-ABs were closely correlated with angiogenic activity, including blood vessel angiogenesis, sprouting, endothelial cell migration, and proliferation (Fig. 3i), whereas mOC-ABs were closely correlated with osteogenic activity, including bone development, mineralization, osteoblast differentiation and ossification (Fig. 3j). In summary, we showed that the proteomic signature and profile similarities between ABs and their corresponding parental cell may result in similar biological functions.

### pOC-ABs and mOC-ABs inherit specific biological functions from their parental cells

**Fig. 4:**
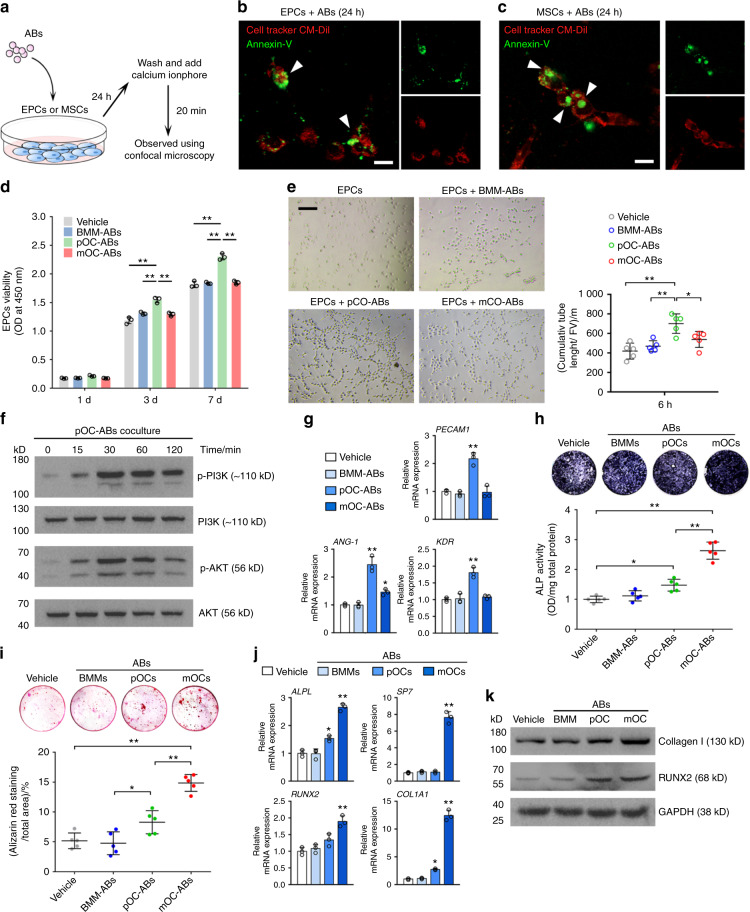
pOC-ABs and mOC-ABs inherit distinct biological functions from their parental cells. **a** Schematic diagram of the AB engulfment assay. Representative confocal micrographs showing cell tracker CM-DiI-labeled (**b**) EPCs or (**c**) MSCs (red) incubated with Annexin-V/FITC-labeled ABs (green). The merged images are shown in the left panel. The white arrows indicate ABs engulfed by recipient cells. The bar represents 10 μm. **d** EPC viability was assessed at 1, 3, and 7 days after treatment with different ABs. “Vehicle” represents EPCs cultured with complete medium. **e** Tube formation assay of EPCs cultured with ABs for 6 h. Representative images (left panel) and quantification analysis of the calculated tube lengths (right panel) are shown. The bar represents 100 μm, *n* = 5. **f** The levels of phosphorylated PI3K and AKT in EPCs were determined by western blot analysis. **g** RT-qPCR analysis of *Pecam1*, *ANG-1*, and *Kdr* expression in EPCs treated with medium containing different ABs. **h** Representative images of ALP staining and quantification of ALP activity. “Vehicle” indicates MSCs cultured with complete medium, *n* = 5. **i** Representative images of ARS staining and quantitative analysis of calcium deposits in MSCs. “Vehicle” indicates MSCs cultured with complete medium, *n* = 5. **j** RT-qPCR analysis of *Alpl*, *Osx*, *Runx2*, and *Col1a1* expression in MSCs cultured with medium containing different ABs. **k** Western blots of the osteogenic markers Collagen I and RUNX2 in MSCs cultured with ABs. The data in the figures are presented as the average ± SD values. Statistically significant differences between the treatment and control groups are indicated as * (*P* < 0.05) or ** (*P* < 0.01)

To validate our bioinformatic predictions, we first investigated the engulfment of ABs by recipient cells. We labeled ABs with Annexin-V/FITC and cultured EPCs or MSCs with the labeled ABs (Fig. 4a). Confocal microscopy was used to observe the internalization of ABs and showed that ABs can be internalized by both EPCs and MSCs (Fig. 4b, c). Analysis of the mean fluorescence intensity showed a constant increase in the Annexin-V intensity in EPCs and MSCs within 24 h (Fig. S5a, d). Western blot analysis was used to evaluate apoptosis after AB engulfment and revealed a lack of cleavage and activation of the apoptosis-associated proteins PARP and CASP9 (Fig. S5b, e). These results showed that EPCs can internalize these three types of ABs without activating apoptotic pathways. Next, we performed a Cell Counting Kit-8 (CCK-8) assay to investigate the proliferation of EPCs cocultured with ABs. Relative to the vehicle, pOC-ABs showed a significant facilitating effect on EPC proliferation, whereas BMM-ABs and mOC-ABs showed no significant effect on EPC viability after 7 d of culture (Fig. 4d). After EPCs were cultured with pOC-AB-containing medium for 24 h, their migration through transwell chamber membranes in response to fetal bovine serum (FBS) was significantly increased relative to that of EPCs cultured with the vehicle (Fig. S5c). Moreover, the tube formation ability of EPCs cultured with media containing different ABs was evaluated; quantification analysis showed that relative to the vehicle, pOC-ABs significantly increased the total tube length of EPCs (Fig. 4e). Western blot analysis showed that phosphorylation of phosphatidylinositol 3-kinase (PI3K) and AKT peaked at 60 min of coculture, indicating that activation of the PI3K/AKT pathway was involved in pOC-AB-promoted EPC proliferation (Fig. 4f). After EPCs were cultured with AB-containing medium for 3 d, the expression of angiogenesis-related mRNAs was evaluated. *Pecam1* and *Kdr* were highly expressed in the pOC-AB group, while the expression of *ANG-1* was promoted in both the pOC-AB and mOC-AB groups compared with the vehicle group (Fig. 4g). Notably, pOCs cannot be completely removed from mOC culture due to the heterogeneity of the cell differentiation rate. Accordingly, mOC-ABs may not be 100% pure and might be contaminated with some pOC-ABs, which may explain why mOC-ABs displayed few promotive effects on EPC migration and differentiation. Collectively, these data demonstrated that pOC-ABs can stimulate EPC proliferation and differentiation. To compare the osteogenic potency among different ABs, we performed Alizarin red S (ARS) staining and alkaline phosphatase (ALP) staining of MSCs cocultured with different ABs. Quantification analysis showed that both pOC-ABs and mOC-ABs but not BMM-ABs have osteogenic potency; however, mOC-ABs exhibited stronger osteogenic potency than pOC-ABs (Fig. 4h, i). RT-qPCR analysis revealed that the osteogenic regulators *Osterix* (*Osx*, also called *Sp7*) and Runt-related transcription factor 2 (*Runx2*), as well as the osteogenic markers *Alpl* and type I collagen (*Col1a1*), were highly expressed in the mOC-AB group (Fig. 4j). Consistent with this finding, western blot analysis confirmed that COL1A1 and RUNX2 in MSCs were upregulated by mOC-AB treatment (Fig. 4k). Collectively, our study indicated that ABs isolated from pOCs and mOCs inherited distinct and specific biological functions from their corresponding parental cells and that pOC-ABs promote angiogenesis, whereas mOC-ABs promote osteogenesis.

### pOC-ABs promote angiogenesis by delivering PDGF-BB to recipient EPCs

**Fig. 5:**
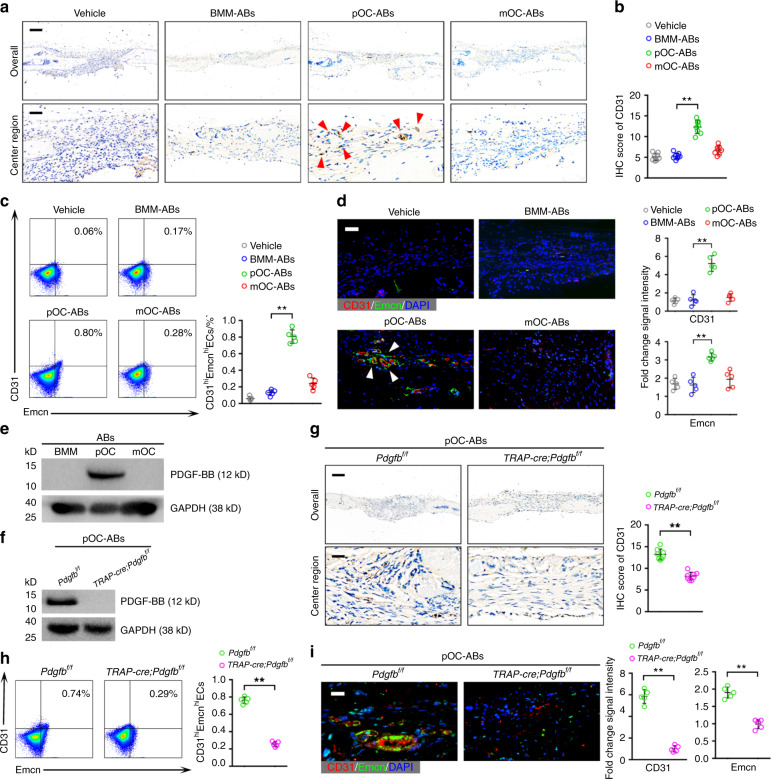
pOC-ABs promote angiogenesis by delivering PDGF-BB to recipient EPCs. **a** IHC of CD31 in mice 2 weeks after engrafting of AB-DBM. The red arrows indicate new CD31-positive vessels formed in the DRA. The bars represent 1 mm (upper) and 100 μm (lower). **b** Semiquantitative analysis showing the IHC score for CD31 in mice grafted with AB-DBM, *n* = 8. **c** Representative flow cytometric analysis with the percentages of CD31^hi^Emcn^hi^ cells among total cells digested from the DRA, *n* = 5. **d** Immunostaining analysis of CD31 and Emcn with quantitative analysis of the relative immunostaining intensities in the DRA. The bar represents 40 μm, *n* = 5. **e** PDGF-BB expression in different ABs, as determined by western blot analysis. As shown, PDGF-BB was included in the proteomic signature of pOC-ABs. **f** PDGF-BB expression in pOC-ABs isolated from pOCs of *TRAP-cre;Pdgfb*^*f/f*^ mice, as determined by western blot analysis. **g** IHC and semiquantitative analysis of CD31 expression in mice grafted with DBM preincubated with *TRAP-cre;Pdgfb*^*f/f*^ pOC-ABs or *Pdgfb*^*f/f*^ pOC-ABs. The bar represents 1 mm in the overview and 100 μm in the center region, *n* = 8. **h** Representative flow cytometric analysis with the percentages of CD31^hi^Emcn^hi^ cells among total cells digested from the DRA, *n* = 5. **i** Representative images of immunostaining of CD31 and Emcn with quantification of the relative CD31 and Emcn immunostaining intensities in the DRA. The bar represents 20 μm, *n* = 5. The data in the figures are presented as the average ± SD values. Statistically significant differences between the treatment and control groups are indicated as * (*P* < 0.05) or ** (*P* < 0.01)

To confirm the proangiogenic effects of pOC-ABs in vivo, a cranial defect mouse model was established. Cranial drilling was conducted, and decalcified bone matrix (DBM) loaded with ABs (AB-DBM) of different origins was implanted in the defect areas 2 weeks before euthanasia. Histological analysis of the defect repair area (DRA) was performed to evaluate angiogenesis. Hematoxylin and eosin (H&E) staining showed that small vessels were markedly enriched in mice grafted with pOC-AB-DBM (Fig. S6a, b). Immunohistochemistry (IHC) of CD31 was performed, and semiquantitative analysis revealed that both male and female mice grafted with pOC-AB-DBM contained more CD31-positive cells than mice treated with the vehicle (Fig. 5a, b; Fig. S6c, d). A recent study identified a specific vessel subtype, a type H vessel, that was highly positive for CD31 and endomucin (CD31^hi^Emcn^hi^) and was shown to couple angiogenesis and osteogenesis in bone remodeling^30^. Intriguingly, we digested cells from the DRA to determine the proportion of CD31^hi^Emcn^hi^ cells and discovered that mice treated with pOC-ABs had the highest proportion of CD31^hi^Emcn^hi^ cells (Fig. 5c). In addition, IF staining of CD31 and Emcn revealed that mice grafted with pOC-AB-DBM had the highest number of CD31^hi^Emcn^hi^ endothelial cells (Fig. 5d). To determine the key factors that govern the proangiogenic ability of pOC-ABs, we focused on PDGF-BB, which can be secreted by pOCs to induce the formation of CD31^hi^Emcn^hi^ endothelial cells^15,31^. Herein, we found that PDGF-BB was included in the proteomic signature of pOC-ABs and was rarely expressed in BMM-ABs or mOC-ABs (Fig. 5e). To investigate whether PDGF-BB contributes to the proangiogenic effect of pOC-ABs, we extracted ABs from pOCs of *TRAP-cre*;*Pdgfb*^*f/f*^ mice, in which PDGF-BB expression is conditionally knocked down in the osteoclast lineage (Fig. 5f). Immunohistochemical analysis of CD31 showed that mice treated with *TRAP-cre;Pdgfb*^*f/f*^ pOC-ABs displayed poor angiogenic ability compared with mice treated with *Pdgfb*^*f/f*^ pOC-ABs (Fig. 5g). Moreover, the number of CD31^hi^Emcn^hi^ endothelial cells was significantly reduced upon knockdown of PDGF-BB in pOC-ABs (Fig. 5h, i). Our findings indicated that PDGF-BB governs the proangiogenic ability of pOC-ABs.

### mOC-ABs promote osteogenesis via RANKL reverse signaling

**Fig. 6:**
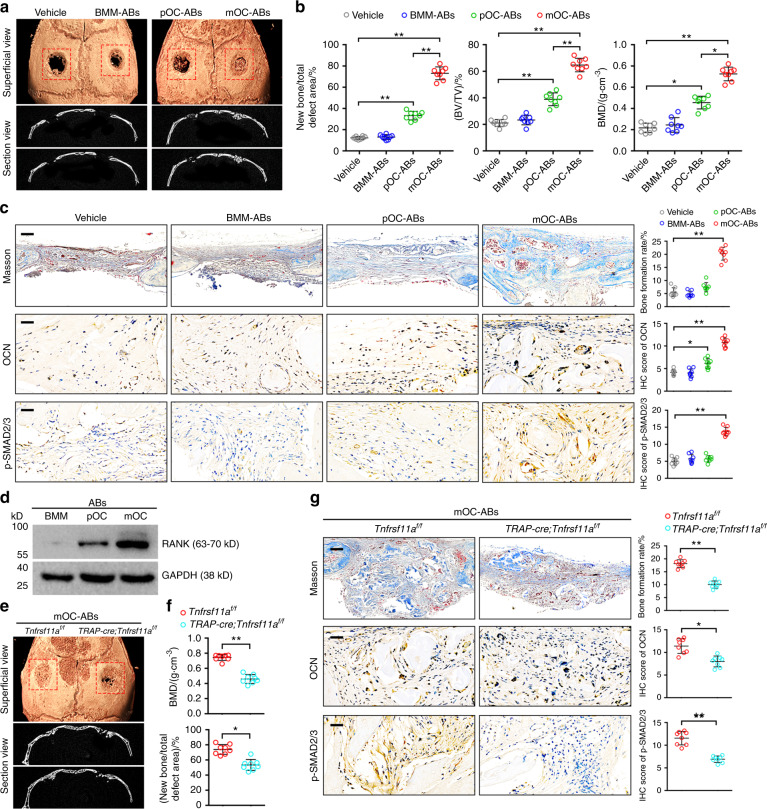
mOC-ABs promote osteogenesis through RANKL reverse signaling. **a** Representative micro-CT images of cranial bone 4 weeks after AB-DBM implantation. **b** Quantification of bone histomorphometric parameters (bone formation ratio, bone volume density (BV/TV), bone mineral density (BMD)) based on micro-CT images, *n* = 8. **c** Representative histological images of Masson staining and IHC of OCN and p-SMAD2/3 in mouse cranial bone sections (left). Quantification of the bone formation ratio and semiquantitative analysis of OCN and p-SMAD2/3 levels in mice grafted with AB-DBM (right). The scale bars represent 300 μm for Masson staining and 70 μm for IHC staining, *n* = 8. **d** Western blot analysis of RANK levels in different ABs. As shown, RANK was included in the proteomic signature of mOC-ABs. **e** Representative micro-CT images of cranial bone treated with *TRAP-cre;Tnfrsf11a*^*f/f*^ mOC-ABs for 4 weeks. **f** Quantification of the amount of new bone formation and BMD in the total DRA of mice treated with *TRAP-cre;Tnfrsf11a*^*f/f*^ mOC-ABs based on micro-CT images, *n* = 8. **g** Representative histological images of Masson staining and IHC of OCN and p-SMAD2/3 in the indicated groups (left). Semiquantitative analysis of OCN and p-SMAD2/3 levels and the bone formation ratio in the indicated groups (right). The scale bars represent 300 μm for Masson staining and 70 μm for IHC staining, *n* = 8. The data in the figures are presented as the average ± SD values. Statistically significant differences between the treatment and control groups are indicated as * (*P* < 0.05) or ** (*P* < 0.01)

To evaluate the osteogenic potency of mOC-ABs in vivo, bone regeneration was assessed in both male and female mice by micro-CT 4 weeks after surgery (Fig. 6a, Fig. S6g). Quantification analysis of the defect areas showed that ABs derived from either pOCs or mOCs markedly enhanced bone repair, while mice treated with mOC-AB-DBM showed stronger osteogenic activity than mice treated with pOC-AB-DBM (Fig. 6b, Fig. S6h). H&E staining showed greater osteoid formation in mice grafted with mOC-AB-DBM than in mice grafted with pOC-AB-DBM (Fig. S6e, f). Consistent with this finding, Masson staining confirmed a significant increase in the bone formation rate in mice implanted with mOC-AB-DBM (Fig. 6c). In addition, IHC of the osteoblast-specific marker osteocalcin (OCN) revealed that mice grafted with mOC-AB-DBM harbored a higher percentage of OCN-positive cells. Consistent with this finding, immunohistochemical staining of calvarial sections showed a similar increase in the p-SMAD2/3 level, suggesting an increased level of osteogenic differentiation. Our previous study indicated that RANK, as a receptor for the osteoclastogenesis-stimulating factor RANKL, also existed in mOC-ABs as vesicular RANK and induced osteogenic differentiation in vitro through RANKL reverse signaling^17^. Consistent with this observation, our LC-MS/MS results herein indicated that RANK was among the most enriched proteins in mOC-ABs, as confirmed by western blot analysis (Fig. 6d). We further extracted ABs from mOCs of *TRAP-cre;Tnfrsf11a*^*f/f*^ mice, in which RANK expression is conditionally knocked down in the osteoclast lineage. Notably, knockout of vesicular RANK abrogated the osteogenic potency of mOC-ABs, as evidenced by hindered bone repair (Fig. 6e, f). Moreover, IHC of calvarial sections showed that mice treated with mOC-ABs displayed decreased bone formation and levels of OCN and p-SMAD2/3 upon RANK knockdown (Fig. 6g). Collectively, these results indicated that engraftment of mOC-AB-DBM facilitated bone formation via vesicular RANK-mediated osteogenic differentiation.

## Discussion

Relative to the other two members of the EV family, i.e., exosomes and MVs^32–34^, much less is known about the roles of ABs in intercellular communication and signal transduction. Although ABs have been reported to contain nucleic acids, proteins and infectious agents^7,8^, few studies have identified the specific AB cargo using high-throughput techniques. We previously determined the whole transcriptome of osteoclast-derived ABs using RNA-seq^17^. Here, to further understand the biological functions of ABs in recipient cells, LC-MS/MS analysis was used to map the proteomic landscapes of osteoclast-derived ABs. To minimize the difference between STS-induced and intrinsically generated ABs, we used a relatively mild dosage of STS to minimize the residual effects and purified the ABs before use to remove all dissolved STS. Bioinformatic analysis revealed that the ABs shared similar proteomic signatures with their parental cells, and this proteomic signature similarity extended to biological similarities, as predicted by GSEA. In vitro studies confirmed the GSEA results, showing that pOC-ABs specifically promoted the angiogenesis of EPCs, whereas mOC-ABs promoted the osteogenesis of MSCs. We established a mouse calvarial bone defect model; DBM loaded with different ABs were grafted into the defect area, and the bone regeneration capability was evaluated. Our data indicated that pOC-ABs specifically increased CD31^hi^Emcn^hi^ endothelial cell formation and that mOC-ABs specifically increased bone mineralization and volume. Combining these findings with those of the mechanistic study, we identified that PDGF-BB was enriched mostly in pOC-ABs and RANK was enriched mostly in mOC-ABs, which determined their featured functions. Notably, our study cannot rule out the possibility that other enriched cytokines may also mediate the biological functions of ABs.

A recent study revealed a new mechanism of AB generation via a ‘beads-on-a-string’ structure and performed proteomic analysis of the ABs formed in this way^3^. The study showed that ABs generated in this way rarely contained nuclear cargo but were rich in proteins involved in cell growth and signal transduction. Similarly, but going one step further, we acquired ABs from the same type of cell at distinct stages and further confirmed the consistency of the ABs with their parental cells in terms of both proteomic signatures and biological functions. In addition, other studies have shown that ABs containing miRNAs regulate signal transduction in recipient cells^35^. Tumor-secreted EVs are also considered key mediators of intracellular crosstalk between cancer cells and adjacent normal cells^36^. Tumor cells overexpress genes such as *EST-1* to drive angiogenesis, and they secrete an abundance of angiogenic factors that promote cancer development^37,38^. EVs secreted from breast cancer cells can extravasate and further facilitate tumor metastasis via the S100/miR-105 regulatory axis^39,40^. The above examples show regulatory patterns similar to those identified in our study, suggesting that cells might continue to regulate and influence other cells via EVs even after apoptosis. Our results demonstrated that ABs from bone cells also have biological functions similar to those of their parental cells, suggesting that this phenotypic inheritance of EVs from parental cells is more general than we previously thought.

**Fig. 7:**
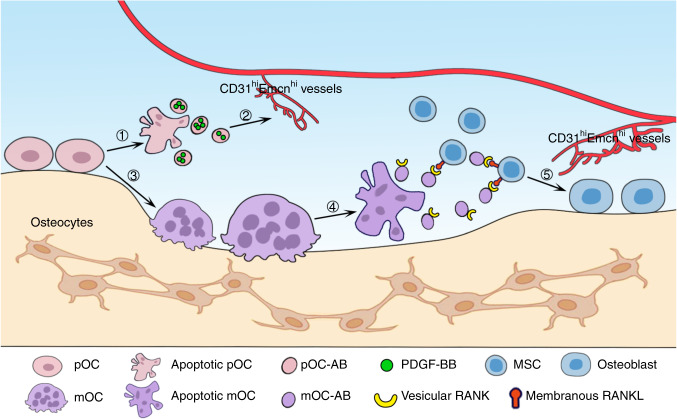
Schematic diagram showing the role of osteoclast-derived ABs in bridging bone resorption and formation in bone remodeling. ① A fraction of pOCs undergo apoptosis and secrete pOC-ABs. ② pOC-ABs induce the formation of CD31^hi^Emcn^hi^ vessels by delivering PDGF-BB to recipient EPCs. ③ pOCs differentiate into mOCs upon stimulation with RANKL and M-CSF. ④ mOCs undergo apoptosis and secrete mOC-ABs at the end of the bone resorption phase. ⑤ mOC-ABs promote osteogenesis through RANKL reverse signaling. In addition, CD31^hi^Emcn^hi^ vessels induced by pOC-ABs improve local nutrient and metabolic waste transport to further maintain the integrity of bone remodeling

Accumulating evidence has revealed the roles of bone cell-generated EVs in bone remodeling^41^. Osteoblast-derived EVs contain specific osteogenic proteins, such as BMP1-7 and ALP, and the noncollagenous matrix proteins OPN and OCN^42^, whereas osteoclast-derived EVs contain proteins that can regulate osteoclast differentiation, such as RANK and RANKL^43^. In addition, evidence has also shown that bone cell-derived EVs can target adjacent cells and mediate intercellular crosstalk. For instance, exosomal miRNAs from osteoclasts and osteocytes have regulatory effects on osteoblast differentiation^24,44^. BMSC-derived exosomes can deliver miR-151-5p to endogenous BMSCs, which rescues the damaged osteogenic ability and decreases the adipogenic ability of these cells^45^. In bone remodeling, the delicate coupling of bone formation and resorption maintains bone homeostasis. On the cellular level, the transition from bone resorption to formation always proceeds in tandem with osteoclast quiescence and apoptosis. Studies have shown that TGF-beta released via osteoclastic bone resorption recruits BMSCs for further osteogenesis^46^. In addition, recent studies have suggested that osteoclast-derived EVs can be delivered to osteoblasts and potentiate osteogenic differentiation^16,17^. Our study showed that pOC-ABs promote angiogenesis by delivering PDGF-BB to recipient EPCs, while mOC-ABs promote osteogenesis via RANKL reverse signaling (Fig. 7). In addition to the well-studied osteoclast-endothelial cell and osteoclast-osteoblast coupling, the involvement of osteoclast-derived ABs further bridges the transition between bone formation and resorption during bone remodeling.

In conclusion, we found that ABs constitute a group of highly biocompatible EVs with proteomic signatures and biological functions largely inherited from their parental cells. Upon engulfment by recipient EPCs and MSCs, osteoclast-derived ABs perform specific regulatory roles determined by the differentiation stage of their parental cells. The regulatory functions of osteoclast-derived ABs expand the concept of cell coupling and potentially bridge the transition between bone resorption and bone formation during bone remodeling.

## Materials and methods

### Cell and reagents

We obtained primary MSCs and BMMs from bone marrow of 11-week-old C57BL/6 mice as previously described^47,48^. After mice were sacrificed, the femurs and tibias were harvested under aseptic conditions. BMCs were collected by washing the medullary cavities of the tibias and femurs. BMCs were then stimulated with 50 ng·mL^−1^ M-CSF for 96 h to obtain BMMs. To isolate MSCs, we cultured BMCs in alpha minimal essential medium (α-MEM; HyClone, USA) for 24 h and then removed the nonadherent cells to obtain MSCs. In addition, we isolated EPCs from mouse peripheral blood as previously described^48^. Cells were maintained in α-MEM supplemented with 10% FBS (BioInd, Israel) and 100 U·mL^−1^ penicillin/streptomycin (Solarbio, Beijing, China) in a 37 °C incubator with 5% CO_2_. We prepared AB media by adding 5 × 10^6^ ABs to 50 mL volumes of complete medium. Purified mouse RANKL and M-CSF (R&D System) were dissolved in α-MEM. An IF staining kit for cytoskeletal staining was purchased from Millipore (Merck KGaA, Germany). A TRAP staining kit was purchased from Solarbio. A FITC-Annexin-V and PI Apoptosis Kit (F6012) was purchased from US Everbright® Inc. (Suzhou, China). Antibodies against PDGF-BB (ab23914) were obtained from Abcam. Antibodies against RUNX2 (bs-1134R), Collagen I (bs-10423R), RANK (bs-7343R), CASP9 (bs-0049R), PARP (bs-2138R), PI3K (bs-10657R), p-PI3K (bs-6417R), AKT (bs-0115M), p-AKT (bs-2720R), CD31 (bs-0468R), Emcn (bs-5884R) and GAPDH (bs-0755R) were purchased from Bioss Antibodies (Beijing, China).

### Osteoclast differentiation assay

BMMs (6 × 10^3^) were incubated in 96-well plates and induced with 100 ng·mL^−1^ RANKL for 0 h, 24 h and 96 h to generate BMMs, pOCs and mOCs, respectively. Osteoclasts at different stages were identified using TRAP staining. The TRAP staining solution was prepared according to the manufacturer’s instructions. Cells were fixed with 4% paraformaldehyde and washed with PBS three times. Then, cells were incubated with the staining solution for 1 h. For IF staining of the cytoskeleton, cells were washed and were then fixed with 4% paraformaldehyde. After permeabilization, cells were incubated with an anti-vinculin antibody (1:500) at room temperature for 60 min. 4′,6-Diamidino-2-phenylindole (DAPI) was then used to stain cell nuclei for 10 min. Finally, the osteoclast surface was observed using a Zeiss LSM-800 microscope.

### Isolation and identification of ABs

For apoptosis induction, cells were treated with 0.5 μmol·L^−1^ STS (MedChemExpress, Shanghai, China) and incubated at 37 °C in 5% CO_2_. After 12 h, cell supernatants were harvested and centrifuged at 300 × *g* for 15 min to remove cell debris. The supernatants were subsequently centrifuged at 3 000 × *g* for 20 min, and the pellets containing ABs were harvested for further experiments. For AB identification, the pellets were stained with Annexin-V/FITC and observed using a Zeiss LSM-800 microscope. For flow cytometry, isolated ABs were incubated with Annexin-V/FITC and PI in the dark for 15 min. After incubation, the samples were pelleted, the supernatant was removed, and the pellets were resuspended in 500 μL of PBS for flow cytometric analysis.

### LC-MS/MS analysis

After isolation and identification, ABs were lysed on ice with RIPA lysis buffer (Beyotime Biotechnology, China), which contained Halt™ Protease Inhibitor Cocktail (Thermo Fisher). Then, the samples were centrifuged at 12 000 × *g* and 4 °C for 15 min to remove subcellular debris. Dithiothreitol (DTT; 1 mol·L^−1^, BIO-RAD) was added to the samples and incubated at 55 °C for 45 min. Subsequently, the protein samples were incubated with 55 mmol·L^−1^ iodoacetamide (IAM; Sigma) solution and reacted in the dark at 37 °C for 30 min. After washing with acetonitrile (Fisher Chemical), 0.02 μg·μL^−1^ trypsin was used for digestion. Peptide segments were separated by reversed-phase high-performance liquid chromatography (HPLC) with an Agilent 300Extend-C18 high-pH column. Finally, the supernatants containing protein peptides were concentrated and freeze dried for LC-MS. Peptides were dissolved in HPLC mobile phase A (0.1 mol·L^−1^ formic acid and 2 mol·L^−1^ acetonitrile) and were then separated in an EASY-NLC 1000 ultra-HPLC system. After this separation, protein peptides were injected into an NSI ion trap for ionization and were then analyzed in a Q Exactive Plus mass spectrometer. The ion trap setting was 2.0 kV. An Orbitrap instrument with high resolution (70 000) was employed for detection and analysis. We deposited the mass spectrometry proteomic data for the ABs in the ProteomeXchange Consortium via the PRIDE partner repository with the dataset. All other data are available from the corresponding author on request. In addition, The raw mass spectrometry data were uploaded to the ProteomeXchange database (http://www.proteomexchange.org) under accession number PXD017245.

### Bioinformatic analysis

We employed RStudio software for data analysis. Protein expression was considered significantly different if the following criteria were met: *P* < 0.05 and FC for DUPs >2.0 or DDPs <0.5. For further analysis, FC values were normalized using log2 ratios. Data visualization, including hierarchical clustering, Venn diagram analysis and volcano plots, was performed as previously described^48^. We generated PCA plots using the ggfortify R package. The subcellular localization of proteins was predicted with WoLF PSORT software. For enrichment analysis, we used clusterProfiler, an R package that automates the classification of BP terms and enrichment analysis of gene clusters^49^. Enrichment analysis of genes encoding proteins in the proteomic signatures of parental osteoclasts was conducted using our previously obtained data (GEO accession number GSE72478). Then, we used the clusterProfiler package for GSEA and visualization.

### In vitro assays of EPC proliferation, migration, and tube formation

We performed a CCK-8 assay to assess cell proliferation and evaluated the cell migration ability using a transwell assay. For the CCK-8 assay, 2 × 10^3^ EPCs were seeded in 96-well plates and incubated with ABs for 1 d, 3 d, and 7 d. According to the manufacturer’s instructions, EPC viability was evaluated using a CCK-8 (HyClone) at 1 d, 3 d, and 7 d. The absorbance of the wells was measured at 450 nm in a 96-well plate reader, and cell viability was evaluated. To test the migration ability of EPCs treated with different ABs, 96-well transwell plates (8 μm pore size, Corning, NY, USA) were used to conduct transwell assays. Cells were cocultured with the three types of ABs for 24 h and were then seeded in the upper chambers with serum-free MEM (1 × 10^4^ cells per well), and DMEM supplemented with 20% FBS was added to the lower chambers. After 24 h of incubation, the upper chambers were wiped carefully to remove nonmigrated cells, and the cells that traversed the membrane were fixed and stained with 0.1% crystal violet. After washing with PBS several times, cells in five random fields per sample were counted. For the tube formation assay, 2 × 10^5^ EPCs were incubated in a 24-well plate, which was precooled and coated with 250 µL of Matrigel (Corning, NY, USA). Cells were then incubated with 500 μL of AB medium or with complete medium as the vehicle. After 6 h, endothelial cells linked by tubes were observed and quantified as previously described^48^.

### In vitro assay for osteogenic differentiation of MSCs

Calcium deposits were observed and quantified under a microscope. For osteogenic differentiation, 1 × 10^6^ MSCs were incubated in 24-well plates and induced with AB-containing osteogenic medium for 14 d and 21 d. After 14 and 21 d of induction, total RNA and protein were harvested for RT-qPCR and western blotting. In addition, ALP activity was assessed using an ALP stain kit (Abcam) after 14 d of induction. For ARS staining, cells induced for 14 d were fixed and stained with 2% ARS (Sigma).

### Preparation of DBM loaded with ABs

DBM was detached from limb bones of cattle and treated as previously described^48^ and was divided into 2.5 mm × 2.5 mm blocks. The blocks were soaked with 75% ethanol for 3 h and rinsed with PBS. Then, ABs were resuspended in fibronectin gel (Corning) at a final concentration of 1 μg·μL^−1^. Subsequently, the gel was added onto the DBM and incubated at 37 °C for 12 h. Finally, the DBM was dried and frozen at −70 °C for further experiments.

### Animal experiments

We obtained *Pdgfb*^*f/f*^ and *Tnfrsf11a*^*f/f*^ mice from The Jackson Laboratory; the source of the *TRAP-cre* mouse strain was previously described^50^. We crossed *TRAP-cre* mice with *Pdgfb*^*f/f*^ and *Tnfrsf11a*^*f/f*^ mice to obtain *TRAP-cre*;*Pdgfb*^*f/f*^ mice and *TRAP-cre*;*Tnfrsf11a*^*f/f*^ mice, respectively. To observe the osteogenic potency of AB-DBM, we established a cranial defect mouse model. In brief, C57BL/6 mice aged 4–6 weeks were anesthetized. A 2 cm incision was made in the center of the head of each mouse to expose the cranium, and a dental drill was used to generate two 2.5 mm defects on both sides of the cranium. After that, AB-DBM was embedded into the bone defects. Thirty-two mice were randomly divided into four groups according to the distinctive types of ABs loaded into the DBM. Cranium samples were obtained 2 weeks and 4 weeks after surgery for further investigation. All animal breeding and experimental procedures were approved by the Institutional Animal Care and Use Committee of Johns Hopkins University School of Medicine.

### IF staining of CD31^hi^Emcn^hi^ vessels

To investigate the expression of CD31 and Emcn in newly formed tissue, we performed IF staining on paraffin sections. In brief, sections were incubated separately with mouse anti-CD31 (1:200, Bioss) and rabbit anti-Emcn (1:200, Bioss) antibodies at 4 °C overnight. Then, sections were incubated with the corresponding fluorophore-labeled secondary antibodies at room temperature in the dark for 1 h. Images of sections were acquired with a Leica TCS SP8 microscope.

### Histological and immunohistochemical evaluation

Mouse cranial bones were collected at 2 weeks (for angiogenesis evaluation) or 4 weeks (for osteogenesis evaluation) after surgery. The process of making bone sections from cranial bones was described in our previous study^48^. After fixation, decalcification, and paraffin embedding, histological sections were cut into 1-μm-thick sections and prepared for subsequent H&E staining. For IHC, the expression of CD31, OCN, and p-SMAD2/3 was detected according to the following procedure. Sections were first incubated separately with rabbit anti-CD31, rabbit anti-OCN, and rabbit anti-p-SMAD2/3 antibodies at 37 °C for 2 h and then with a biotinylated secondary antibody (diluted in 1% BSA-PBS). After a DBA Chromogenic Kit (Ybscience) was used for chromogenic staining, the stained sections were washed and observed using light microscopy. The German semiquantitative method was used to evaluate the IHC scores; the specific scoring criteria were explained in previous studies^48^.

### Statistical analysis

The data are presented as the mean ± standard deviation (SD) values. Student’s *t* test was employed to compare two individual groups. One-way ANOVA followed by the Student-Newman-Keuls post hoc test was employed to compare multiple sets of data. Data analyses were repeated at least three times. *P* < *0.05* was considered statistically significant, and significance is indicated as *P < 0.05 and **P < 0.01.

## Supplementary information

Supplement (for review only)

Osteoclast-derived apoptotic bodies bridge bone resorption and formation in bone remodeling
