# Supplementary material for: Osteoclast-derived apoptotic bodies couple bone resorption and formation in bone remodeling
Source: Bone Res. 2021 Jan 11;9:5. doi: 10.1038/s41413-020-00121-1 (PMC7801485; doi:10.1038/s41413-020-00121-1)
Supplement: Supplementary file 1 — Supplement (for review only) [file 41413_2020_121_MOESM1_ESM.pdf]

Manuscript Title:

Osteoclast-derived apoptotic bodies bridge bone resorption and formation in bone remodeling

7,030 words – 95 matches – 39 sources

Similarity Index: 14%

- **Instances of plagiarism are highlighted in different colors.**
- **Color-coded highlighting corresponds to matching sources in the CrossRef database.**

CC_200908G700

WORD COUNT 7030 TIME SUBMITTED 08-SEP-2020 09:34PM
PAPER ID 62947751

Osteoclast-derived apoptotic bodies bridge bone resorption and formation in bone remodeling

Keywords: Apoptotic body, Osteoclast, Vesicle bioinformatics, Intercellular communication

Abstract

Bone remodeling is precisely coordinated by bone resorption and formation. Apoptotic osteoclast generates huge amounts of apoptotic bodies (ABs) marking the end of bone resorption phase, whereas the functions of osteoclast derived ABs remain largely unknown. Here we identified the molecular profiling of ABs from osteoclasts of distinct differentiation stages and investigated their corresponding functions. ABs were isolated from apoptotic bone marrow macrophages (BMMs), pre-osteoclasts (pOCs) and mature osteoclasts (mOCs) induced by staurosporine. Proteomic signature analyzed with liquid chromatography-tandem mass spectrometry (LC-MS/MS) suggested remarkable protein cargo differences of different ABs. Further bioinformatics analysis showed that proteomic signatures of ABs exhibited high similarities with their parental cells. Functionally, pOC-ABs induced endothelial progenitor cell (EPC) differentiation and increased $CD31^{hi}Emcn^{hi}$ endothelial cell formation in a murine bone defect model via PDGF-BB. mOC-ABs induced osteogenic differentiation of mesenchymal stem cells (MSCs) and facilitated osteogenesis via RANKL reverse signaling. Together, our results mapped the detailed proteomic landscapes of ABs derived from osteoclasts and showed their potential biological roles are important in coupling bone formation with resorption in bone remodeling.

Introduction

Billions of cells go through apoptosis every day to maintain the physiological homeostasis of human body.[1] At the late stage of apoptosis, the nucleus and cytoplasm of apoptotic cells compact and disassemble into subcellular membrane-bound extracellular vesicles named as apoptotic bodies (ABs).[2,3] As a subset of extracellular vesicles (EVs), ABs (1-5 µm) are much bigger than exosomes (100-200 nm) or microvesicles (MVs) (100-1000 nm) and are only generated by apoptotic cells.[4] The assembly of organelles such as endoplasmic reticulum, nuclear contents and mitochondria into ABs is regarded as a random process,[5,6] therefore the contents of ABs may contain various materials such as proteins, lipids and RNAs.[7,8] Several autoantigens, such as complements (C1QC and C3B) and histone family (especially histone 2B and histone 3)were found to be highly enriched in ABs and were therefore commonly regarded as markers to differentiate ABs with other subtypes of EVs.[9,10] Traditional perspectives view ABs as a group of garbage bags that encapsulate remanent fragments of dead cells and are subsequently phagocytized to prevent adverse impacts on the surroundings. However, increasing evidences are supporting participation of ABs in biological events including inflammation, autoimmune and cancer via regulating recipient cells.[11,12] These findings demonstrated that ABs are not only cell debris but also involved in intercellular crosstalks. However, studies revealing the content in detail within ABs are lack and the detailed mechanism of AB biological roles remains unknown.

Osteoclasts are bone resorbing cells which play important roles in bone remodeling and metabolism.[13] Activated by two critical factors namely nuclear factor κB ligand (RANKL) and macrophage-colony stimulating factor (M-CSF), macrophage progenitor cells differentiated into mononuclear pOCs, and multiple pOCs further fuse together to differentiate multinucleated mOCs.[14] Although both are called osteoclasts, the functions of pOC and mOC are quite different. pOCs generally do not show a significant bone resorption function but can secrete anabolic cytokines, which mediate intercellular crosstalks with EPCs to promote angiogenesis by releasing PDGF-BB.[15] mOCs on the other hand, in addition to the bone resorption activity, showed strong potency of promoting osteogenesis through RANKL reverse signaling.[16,17] Our previous studies also showed that pOC and mOC have distinct signatures of transcriptome and small RNAs.[18] *In vivo* osteoclasts have a relative short lifespan of only few weeks before apoptosis.[19,20] In bone remodeling, osteoclasts undergo apoptosis at the end of bone resorption phase and produce huge amounts of ABs, followed by osteoblasts moving into the resorption space, indicating the initiation of bone formation stage.[21,22] Previous findings have centred around the process in which osteoclasts couple with osteoblasts by releasing cytokines, secretory proteins and exosomes.[23-25] However, the functions of osteoclast-derived ABs on surroundings and cell coupling remain unclear.

Here we plotted the signatures of the whole-proteome in osteoclast-derived ABs. Through bioinformatics analysis, we displayed remarkable protein cargo differences among different ABs and revealed respective proteomic signatures. By comparing with the whole-proteome of

respective parental cells, a high similarity of signatures was detected. This similarity was further confirmed on the functional level via *in vitro* and *in vivo* tests.

Results

Isolation and characterization of ABs from BMMs, pOCs and mOCs

We isolated ABs derived from osteoclasts at different stages according to the outlined experimental design (Fig. 1a). Whole bone marrow cells, derived from the hind limbs of 11-week old male mice were stimulated with M-CSF for 48 h to generate BMMs. BMMs were then induced to pOCs (24h after RANKL and M-CSF stimulation) and mOCs (96 h after RANKL and M-CSF stimulation) validated by tartrate resistant acid phosphatase (TRAP) stain and immunofluorescent (IF) stain of cytoskeleton (Fig. 1b). Quantitative analysis showed that TRAP-positive cells at 24h exceeded 80% of total cells and almost reached 100% at 96 h, while actin ring-positive multinucleated cells (more than three nuclei) account for less than 3% at 24h but increased to 8% at 96h (Fig. 1c). The gene array analysis showed a remarkable increase of most RANKL-dependent gene expression in pOC and mOC confirming the distinct stages (Fig. 1d). ABs from BMMs, pOCs and mOCs were isolated after cell apoptosis induceing by staurosporine (STS).[26] Cells undergoing apoptosis were characterized by reduction of cytoplasmic refraction and obvious membrane blebbing (Fig. S1). After apoptosis induction, differential centrifugation was used to separate AB-sized EVs from dead cells, cell debris and other small EVs.[27] For AB identification, we stained ABs with Annexin-V-FITC and observed using confocal microscopy; results showed that ABs were characterized as spherical vesicles, with monolayer membranes and positive for Annexin-V (Fig. 1e). We next used flowcytometry to detect the purity of seperated ABs (Fig. 1f). Forward/side scatter (FSC/SSC) analysis showed a significant difference between ABs and cells in size. Annexin-V/FITC analysis showed that ABs express intermedium level of phosphatidylserine (PS) relative to apoptotic cells, whereas viable cells barely showed PS exposure. PI staining also identified a small AB subset is positive for PI, implying that a small fraction of ABs, but not all, contains nuclear DNA. No significant differences were observed in size and Annexin-V intensity among different ABs.

Proteomic profiling of ABs derived from BMMs, pOCs and mOCs

To establish the proteomic landscape of osteoclast-derived ABs, AB samples were lysed and digested for LC-MS/MS analysis. Raw data were processed using MaxQuant software with the database setting to Mouse_Swissprot_1808.[28] In total, 25,623 peptides and 4,306 proteins were detected and corresponding proteomic profile of three ABs was presented as heatmap after analyzed using hierarchical clustering (Fig. 2a). Among them, 3,020, 3,301 and 3,720 proteins were identified in ABs derived from BMM, pOC and mOC, VENN analysis further revealed intersected and distinct protein signatures in different ABs (Fig. 2b). Three comparison groups were established as pOC-ABs *vs* BMM-ABs, mOC-ABs *vs* BMM-ABs,

and mOC-ABs *vs* pOC-ABs. All the differentially expressed proteins (DEPs) were statistically significant ($p < 0.05$) with fold-change (FC) greater than 2.0. The distribution of DEPs in three comparison groups was shown (Fig. S2a). These DEPs were mainly associated with cellular components including cytoplasm, nucleus and mitochondria (Fig. 2c). All DEPs among three groups were determined and visualized in the volcano plots (Fig. 2d).

To better understand the function of AB protein cargo, we further divided the DEPs into differentially upregulated proteins (DUPs, FC> 2.0) and differentially downregulated proteins (DDPs, FC< 0.5). For DUPs, relative to BMM-ABs, the number of DUPs from cytoplasm in pOC-ABs (33.8%) is almost equal to that from nucleus (31.2%), while the number of DUPs from cytoplasm in mOC-ABs (44.3%) is significantly higher than that from nucleus (20.5%) (Fig. S2b, d); relative to pOC-ABs, the DUPs from mitochondria in mOC-ABs (22.8%) increased significantly, even more than proteins from the nucleus (21.1%) (Fig. S2f). For DDPs, compared with BMM-ABs, the number of DDPs from nucleus in pOC-ABs (38.7%) and mOC-ABs (41.1%) was significantly higher than that from cytoplasm (24.2% and 24.9%) (Fig. S2c, e). These results suggested a significant difference in subcellular localization of DEPs among three ABs. Briefly, BMM-ABs have a higher abundance of nuclear proteins whereas pOC-ABs contain more cytoplasmic proteins, and mOC-ABs are rich in mitochondrial proteins. GO enrichment analysis showed a predicted functions of DEPs in these ABs. GO terms were categorized into biological process (BP), cellular component (CC) and molecular function (MF) and ranked by enrich factors (-log10[pvalue]). Relative to BMM-ABs, DUPs in pOC-ABs were enriched in BP terms correlated with cell differentiation and development and DDPs in pOC-ABs were enriched in cell response to external stimulus (Fig. S3a, b), whereas DDPs in mOC-ABs were mainly enriched in immune system development and immune response (Fig. S3d). Relative to pOC-ABs, DUPs in mOC-ABs were mainly enriched in cell metabolism, while DDPs in mOC-ABs were mainly associated with cellular transport (Fig. S3e, f). Basically, the differences may be caused by the different functions of parental cells, for pOC is still active of differentiation during osteoclastogenesis, whereas mOC lost most of the immunological characteristics and properties compared with monocytes/macrophages.[29] These results drew a proteomic map of three ABs and suggested distinct biological functions of different ABs.

Resemblances of proteomic signatures of ABs with parental cells indicates functional similarities

To further clarify the specific functions of different ABs, we performed VENN analysis of DEPs to screen the specific AB proteomic signatures (Fig. 3a). In this way, we identified 28, 23 and 71 specific signatures displayed in ABs derived from BMMs, pOCs and mOCs respectively, which were subsequently presented as heatmap using hierarchical clustering analysis (Fig. 3b). Principal-components analysis (PCA) was performed to detect the signature-to-signature distance revealing a significant separated clustering among the

signatures of different ABs (Fig. 3c). GO enrichment and subcellular structure localization analysis of AB protein signatures further revealed the differences are in both functions and components (Fig. S4). We then used our previously reported gene expression data of BMM, pOC and mOC to identify the relationship between ABs and corresponding parental cells.[18] Intriguingly, of the 122 identified AB signatures, most of the candidate genes were also found to be highly expressed in corresponding parent cells. VENN analysis showed that AB protein signature overlaps significantly more with corresponding parental cells (5.5%-9.6%) relative to non-parental cells (0.8%-1.1%) (Fig. 3d). Then we asked whether the overlaps of protein signatures reflect a parental cell-like profile by performing gene set enrichment analysis (GSEA). Results showed that signatures of both pOC-ABs and mOC-ABs exhibit highly and specific similarities relative with the parental cells (Fig. 3e, f). To test if the proteomic signature and profile resemblances between AB and the parental cell may result in functional similarities, we performed GO analysis and the most enriched GO terms in pOC-ABs are associated with angiogenic activities while mOC-AB are more enriched in osteogenesis terms (Fig. 3g, h). We then assessed AB functions by GSEA on selected predefined gene sets of biological processes that are known specific to the parental cells. Intriguingly, GSEA results showed that pOC-ABs were closely correlated with angiogenic activity including blood vessel angiogenesis, sprouting, endothelial cell migration and proliferation (Fig. 3i); whereas mOC-ABs were closely correlated with osteogenic activity including bone development, mineralization, osteoblast differentiation and ossification (Fig. 3j). Together, we showed that the proteomic signature and profile resemblances between AB and the parental cell may result in similar biological functions.

pOC-ABs and mOC-ABs inherited specific biological functions from the parental cells

To validate our bioinformatics predictions, we first investigated the engulfment of ABs by recipient cells. We labeled ABs with Annexin-V/FITC, and cultured EPCs or MSCs with labeled ABs (Fig. 4a). Confocal microscopy was used to observe the internalization of ABs, the results showed that ABs can be internalized by both EPCs and MSCs (Fig. 4b, c). Mean fluorescence analysis showed a constant increasing of Annexin-V intensity in EPCs and MSCs within 24h (Fig. S5a, d). Western blot analysis was used to evaluate cell apoptosis after ABs engulfment, revealed a lack of cleavage and activation of the apoptosis-associated proteins PARP and CASP9 (Fig. S5b, e). These results showed that EPCs can internalize three ABs without activating apoptotic pathways. After that, we performed CCK-8 assay to investigate the proliferation of EPCs co-cultured with ABs. The results revealed that pOC-ABs showed a significant facilitating effect on EPC proliferation relative to the vehicle, whereas BMM-ABs and mOC-ABs showed no significant change in EPCs viability after 7 days of culture (Fig. 4d). After culturing with pOC-AB-media for 24 hours, EPCs migration through transwell chambers significantly increased in response to fetal bovine serum relative to the vehicle (Fig. S5c). Tube formation ability of EPCs cultured with different AB-media

was also detected; quantification analysis showed that pOC-ABs significantly enhanced the cumulative tube length of EPCs relative to the vehicle (Fig. 4e). Western blot analysis showed that phosphorylation of phosphatidylinositol 3-kinase (PI3K) and AKT was peaked at 60 min after co-culture, indicating that activation of PI3K/AKT pathway was involved in pOC-ABs promoted EPC proliferation (Fig. 4f). After EPCs were cultured with AB-media for 3 days, angiogenesis-related mRNA expression was detected. The results revealed that *Pecam1* and *Kdr* were highly expressed in pOC-ABs groups, while the expression of *ANG-1* was promoted in both pOC-ABs groups and mOC-ABs groups compared with the vehicle (Fig. 4g). It is worth noting that pOCs cannot be completely removed in mOCs culture due to the heterogeneity of cell differentiation rate. Accordingly, mOC-ABs may not be 100% pure and might be doped with some pOC-ABs, which may be the reason mOC-ABs also displayed few promoted effects on EPC migration and differentiation. Together, these data demonstrated that pOC-ABs have stimulatory capacity for EPC proliferation and differentiation. To compare the osteogenic potency among different ABs, we performed alizarin red staining and alkaline phosphatase (ALP) staining of MSCs co-cultured with different ABs. Quantification analysis showed that both pOC-ABs and mOC-ABs have osteogenic potency but not BMM-ABs; however, mOC-ABs exhibited a stronger potency of osteogenic relative to pOC-ABs (Fig. 4h, i). RT-qPCR analysis revealed that osteogenic regulators *Osterix* (also known as *Sp7*) and Runt-related transcription factor 2 (*Runx2*), as well as osteogenic markers *Alpl* and type I collagen (*Col1a1*) were highly expressed in mOC-ABs groups (Fig. 4j). Consistently, Western Blot analysis confirmed the upregulation of COL1A1 and RUNX2 by mOC-ABs treatment in MSCs (Fig. 4k). Collectively, our study indicated that ABs isolated from pOCs and mOCs inherited distinct and specific biological functions from the corresponding parental cells, pOC-ABs favors angiogenesis whereas mOC-ABs favors osteogenesis.

pOC-ABs promote angiogenesis via delivering PDGF-BB to recipient EPCs

To confirm the pro-angiogenesis effects of pOC-ABs *in vivo*, cranial defect mice model was made. Cranial drilling was conducted and DBM loaded with ABs of different origins was implanted in the defect areas two weeks before euthanasia. Histological analysis of defect repair area (DRA) for angiogenesis evaluation was performed. H&E staining found that the small vessels were remarkably enriched in mice grafted with pOC-AB-DBM (Fig. S6a, b). Immunohistochemistry (IHC) of CD31 was performed and semi-quantitative analysis revealed that both male and female mice grafted with pOC-AB-DBM contained more CD31 positive cells compared with the vehicle (Fig. 5a, b, Fig. S6c, d). Recent study identified a specific vessel subtype as type H vessel that is highly positive for CD31 and endomucin ($CD31^{hi}Emcn^{hi}$), has been shown to couple angiogenesis and osteogenesis in bone modeling.[30] Intriguingly, we digested cells from DRA to detect the proportion of $CD31^{hi}Emcn^{hi}$ cell and discovered that mice treated with pOC-ABs have the highest

proportion of CD31^{hi}Emcn^{hi} cells (Fig. 5c). Immunofluorescent staining of CD31 and Emcn also revealed that the number of CD31^{hi}Emcn^{hi} endothelial cells of mice grafted with pOC-AB-DBM was the strongest (Fig. 5d). To determine the key factors that govern the pro-angiogenic ability of pOC-ABs, we focused on PDGF-BB, which can be secreted by pOCs to induce the formation of CD31^{hi}Emcn^{hi} endothelial cells.[15,31] Herein, we found that PDGF-BB is among the proteomic signatures of pOC-ABs, and is rarely expressed in BMM-ABs or mOC-ABs (Fig. 5e). To investigate if PDGF-BB contributes to the pro-angiogenic effect in pOC-ABs, we extracted ABs from pOCs of *TRAP-cre;Pdgfb$^{f/f}$* mice in which PDGF-BB expression is conditionally knocked down in osteoclast lineage (Fig. 5f). Immunohistochemical analysis of CD31 showed that mice treated with *TRAP-cre;Pdgfb$^{f/f}$* pOC-ABs displayed poor angiogenesis ability compared with *Pdgfb$^{f/f}$* pOC-ABs treatment (Fig. 5g). Moreover, CD31^{hi}Emcn^{hi} endothelial cell number showed a significant reduction upon knockdown of PDGF-BB in pOC-ABs (Fig. 5h, i). Our findings indicated that PDGF-BB determines the pro-angiogenic ability of pOC-ABs.

mOC-ABs promote osteogenesis via RANKL reverse signaling

For osteogenic potency evaluation of mOC-ABs *in vivo*, bone regeneration was assessed in both male and female mice by micro-CT 4 weeks after surgery (Fig. 6a, Fig. S6g). Quantification of defect areas showed that ABs derived from both pOC and mOC markedly enhanced bone repair, while mice treated with mOC-AB-DBM showed the stronger osteogenic activity (Fig. 6b, Fig. S6h). H&E staining showed more osteoid formation in mice grafted with mOC-AB-DBM (Fig. S6e, f). In consistency, Masson staining confirmed significant increase of bone formation rate in mice implanted with mOC-AB-DBM (Fig. 6c). In addition, IHC of osteoblast specific marker osteocalcin (OCN) revealed that mice grafted with mOC-AB-DBM showed a higher percentage of OCN positive cells. Consistently, IHC results of calvarial section showed a similar increased expression of p-SMAD2/3 suggesting an increased osteogenic differentiation level. Our previous study proved that RANK, as receptor for osteoclastogenesis stimulating factor RANKL, also existed in mOC-ABs as vesicular RANK and induced osteogenic differentiation *in vitro* through RANKL reverse signaling.[17] Herein our LC-MS/MS results also indicated that RANK is one of the most enriched protein in mOC-ABs, confirmed by western blot analysis (Fig. 6d). We further extracted ABs from mOCs of *TRAP-cre;Tnfrsf11a$^{f/f}$* mice in which RANK expression is conditionally knocked down in osteoclast lineage. Notably, knockout of vesicular RANK abrogated the osteogenic potency of mOC-ABs characterized by hindered bone repair (Fig. 6e, f). Moreover, IHC results of calvarial section showed that mice treated with mOC-ABs displayed decreased bone formation, OCN and p-SMAD2/3 expressions upon RANK knock down (Fig. 6g). Together, these results indicated that mOC-AB-DBM implantation facilitated bone formation via vesicular RANK mediated osteogenic differentiation.

Discussion

Relative to the other two members of EV family exosomes and microvesicles,[32-34] ABs raise much less awareness of the role in intercellular communication and signal transduction. Although it is reported that ABs contained nucleic acids, proteins and infectious agents,[7,8] few studies reported the detailed AB cargo using high throughput techniques. We previously showed the whole transcriptome of osteoclast-derived ABs using RNA-seq.[35] To further understand the biological functions of ABs on the recipient cells, LC-MS/MS analysis was used to map the proteomic landscapes of osteoclast-derived ABs. To minimize the difference between STS and intrinsically generated ABs, we used a relatively mild dosage of STS to minimize the residual effects and purified the apoptosis bodies before use to remove all the dissolved STS. Bioinformatics analysis revealed that ABs share similar proteomic signatures with the parental cells, and this proteomic signature resemblance transited to biological similarities predicted by GSEA. *In vitro* studies confirmed the GSEA results showing that pOC-ABs specifically promoted angiogenesis of EPCs, whereas mOC-ABs specifically promoted osteogenesis of MSCs. We established a mouse calvarial bone defect model and DBMs loaded with different ABs were implanted into the defect area and evaluated the bone regeneration. Our data indicated that pOC-ABs specifically increased $CD31^{hi}Emcn^{hi}$ endothelial cell formation and mOC-ABs specifically increased bone mineralization and volume. Combined with the mechanistic study, we identified PDGF-BB is mostly enriched in pOC-ABs and RANK is mostly enriched in mOC-ABs which determined their featured functions. Of note, our study cannot rule out the possibility that other enriched cytokines may also be engaged in the biological functions of ABs.

A recent study revealed a new mechanism of AB generation via a 'beads-on-a-string' pattern and analyzed the ABs formed in this way on the proteomic level.[3] They found that ABs generated in this way contain rare nuclear contents, but were rich in proteins involved in cell growth and signal transduction. Similarly but one step further, we acquired ABs from the same type of cell at distinct stages and further confirmed the consistency of AB with the parental cell in both proteomic signatures and biological functions. Besides, studies also showed that ABs containing miRNA regulate signaling transduction in recipient cells.[36] Tumor secreted EVs are also regarded as key mediators of intracellular crosstalks between cancer cells and adjacent normal cells.[37] It is already known that tumor cells overexpress genes like *EST-1* to drive angiogenesis, and secrete abundant angiogenic factors that conduced to cancer development.[38,39] EVs secreted from breast cancer cells are capable of extravasation and further facilitate tumor metastasis via S100/miR-105 regulatory axis.[40,41] The above examples showed similar regulatory pattern relative to our study suggesting that cells might continue to regulate and exert influences on other cells via EVs even after cell apoptosis. Our results demonstrated that ABs from bone cells also have biological functions similar with the parental cells, suggesting that this phenotype inheritance of EVs from parental cell is more general than we previously thought.

Increasing evidences are revealing the roles of bone cell-generated EVs in bone remodeling.[42] It is found that osteoblast-derived EVs contain specific proteins osteogenic such as BMP1-7, ALP and non-collagenous matrix protein OPN, OCN,[43] whereas osteoclast-derived EVs contain proteins, which can regulate osteoclast differentiation such as RANK and RANKL.[44] Besides, evidences also showed that bone cell-derived EVs can target adjacent cells and mediate intracellular crosstalks. For instance, exosomal miRNAs from osteoclasts and osteocytes have regulatory effects on osteoblast differentiation.[24,45] BMSCs-derived exosomes can deliver miR-151-5p to endogenous BMSCs, which rescued damaged osteogenic ability and decreased adipogenic ability[46] In bond remodeling, the delicate coupling of bone formation and resorption maintains bone homeostasis. On the cellular level, the transition from bone resorption to formation always proceeds along with the quiescence and apoptosis of osteoclasts. Studies showed that TGF-beta released by osteoclastic bone resorption recruit BMSCs for further osteogenesis.[47] Recent studies suggested that osteoclast-derived EVs can be delivered to osteoblasts and potentiate osteogenic differentiation.[16,17] In our study, we showed that pOC-ABs promote angiogenesis via delivering PDGF-BB to recipient EPCs while mOC-ABs promote osteogenesis via RANKL reverse signaling (Figure 7). Other than the well-studied osteoclast-endothelial and osteoclast-osteoblast cell coupling, the involvement of osteoclast-derived ABs further bridged the transition between bone formation and resorption in bone remodeling.

In conclusion, we found that apoptotic body is a group of highly biocompatible EVs with proteomic signatures and biological functions largely inherited from the parental cells. Upon engulfment by recipient EPCs and MSCs, osteoclast-derived apoptotic bodies showed specific regulatory roles determined by parental cell differentiation stages. The regulatory functions of osteoclast-derived apoptotic bodies expand the concept of cell coupling and potentially bridge the transition of bone resorption and bone formation in bone remodeling.

Materials and methods

Cell and reagents

We obtained primary MSCs and BMMs from 11-week-old C57BL/6 mice bone marrow as previously described.[48,49] After mice were sacrificed, mouse femur and tibia were dissected under aseptic conditions. Bone marrow cells (BMCs) were collected through washing the medullary cavity of tibia and femur. BMCs were then stimulated with 50 ng/ml M-CSF for 96 hours to acquire BMMs. For MSCs separation, we cultured BMCs in alpha minimal essential medium (α-MEM; Hyclone, USA) for 24 hours and then removed the non-adherent cells to obtain MSCs. Besides, we also separated EPCs from mice peripheral blood as previously described.[49] Cells were maintained in α-MEM supplemented with 10% fetal bovine serum (BioInd, Israel) and 100 units/ml penicillin/streptomycin (Solarbio, Beijing, China) in a 37℃ incubator with 5% CO_2. We prepared AB-media via adding 5×10^6 ABs into every 50 ml of complete medium. Purified mouse RANKL and M-CSF (R&D System) were

dissolved in α-MEM. Immunofluorescent (IF) Staining Kit for cytoskeleton was purchased from Millipore (Merck KGaA, Germany). TRAP-kit was purchased from Solarbio. FITC-Annexin V and PI Apoptosis Kit (F6012) was purchased from US Everbright® Inc. (Suzhou, China). Antibodies against histone 3 (bs-17422R), CD9 (bs-2489R), CASP9 (bs-0049R), PARP (bs-2138R), PI3K (bs-10657R), p-PI3K (bs-6417R), AKT (bs-0115M), p-AKT (bs-2720R), CD31 (bs-0468R), endomucin (bs-5884R) and GAPDH (bs-0755R) were purchased from Bioss Antibodies (Beijing, China).

Osteoclast differentiation assay

6×10^3 BMMs were incubated in 96-well plates and induced with 100 ng/ml RANKL for 0h, 24h and 96h to generate BMMs, pOCs and mOCs, respectively. Osteoclasts at different stage were identified using tartrate-resistant acid phosphatase (TRAP) stain. TRAP stain solution was prepared according to the manufacturers' instructions. Cells were fixed with 4% paraformaldehyde and washed with PBS for three times. After that, cells were incubated with staining solution for 1 hour. For immunofluorescent (IF) staining of cytoskeleton, cells were wash and fixed in 4% paraformaldehyde. After permeabilization, cells were incubated with anti-vinculin (1:500) at room temperature for 60 min. 4′,6-diamidino-2-phenylindole was then used to stain cell nuclei for 10 min. Finally, osteoclast surface was observed using Zeiss LSM-800.

Isolation and identification of ABs

For apoptosis induction, 5×10^6 cells were treated with 5μM STS and incubated at 37°C with 5% CO_2. After 12 hours, the supernatants of cells were harvested and centrifuged at $300\times g$ for 15 min to eliminate cell fragments. The supernatants were subsequently centrifuged at $3000\times g$ for 20 minutes, and the pellets containing ABs were harvested for further experiments. For ABs identification, the pellets were stained with Annexin V-FITC and observed using ZEISS LSM-800. For flowcytometry, separated ABs were incubated with Annexin V-FITC and PI in dark for 15 min. After incubation, the samples were pelleted to remove the supernatant, and re-suspended in 500μL PBS for flowcytometry analysis.

LC-MS/MS analysis

After isolation and identification, ABs were lysed on ice with RIPA lysis buffer (Beyotime Biotechnology, China), which contained with Halt™ Protease Inhibitor Cocktail (Thermo Fisher). Then samples were centrifuged at $12{,}000\times g$ and 4 °C for 15 min to remove subcellular debris. Samples were added with 1M DTT (BIO-RAD) and incubated at 55 °C for 45min. Subsequently, protein samples were incubated with 55 mM IAM (Sigma) solution and reacted in darkness at 37 °C for 30 min. After washing with acetonitrile (Fisher Chemical), 0.02ug/uL trypsin was used for digestion. Peptide segments were graded by reverse HPLC with a high pH column Agilent 300Extend C18. Finally, the supernatant containing protein

peptides were concentrated and freeze drying for LC-MS. The peptides were dissolved in liquid chromatography (HPLC) mobile phase A (0.1M formic acid and 2 M acetonitrile) and then separated using an EASY-NLC 1000 ultra-high performance liquid system. After separation with ultra-high performance liquid system, protein peptides were injected into an NSI ion trap for ionization and then analyzed by Q Exactive Plus mass spectrometry. The ion trap settings were 2.0 kV. Orbitrap instrument with high resolution (70,000) was employed detection and analysis. The mass spectrometry proteomics data have been deposited to the ProteomeXchange Consortium via the PRIDE partner repository with the dataset. All other data are available from the corresponding author on request. The raw data of mass spectrometer have been uploaded to ProteomeXchange database (http: // www. proteomexchange. org) with identifier PXD017245.

Bioinformatics analysis

We employed Rstudio software for data analysis. The expression of protein was considered significant difference with $p < 0.05$ and a FC (DUPs > 2.0, DDPs < 0.5). For further analysis, the FC was normalized using log2 ratios. Data visualization including hierarchical clustering, VENN analysis and volcano plots were performed as previously described. [49] We generated principal component analysis (PCA) plots using ggfortify R package. Protein subcellular localization prediction was conducted with wolfpsort soft. For enrichment analysis, we used clusterProfiler, an R package that automatize the classification of BP terms and the enrichment analysis of gene clusters.[50] Enrichment of genes, which encode proteomic signatures of parental osteoclasts were conducted using our previously data (GEO repository, accession number GSE72478). Afterwards, we used clusterProfiler package for GSEA and visualization.

In vitro assay for proliferation, migration and tube formation of EPCs

We performed CCK-8 assay to assess cell proliferation, and evaluated cell migration ability using transwell assay. For CCK-8 assay, 2×10^3 EPCs were seeded in 96-well plates and incubated with ABs for 1d, 3d and 7d. According to the manufacturer's instruction, EPCs viability was evaluated using Cell Counting Kit-8 (Hyclone) at 1d, 3d and 7d. Cell absorbance was measured by a 450 nm 96-hole plate reader and cell viability was evaluated. To test the migration ability of EPCs treated with different ABs, transwell-96 well plates (8μm, Corning, NY, USA) were used to conduct transwell assay. Cells were co-cultured with three ABs for 24h follow by seeding in the upper chamber with serum-free MEM (1×10^4 per well), and the lower chamber was added with DMEM supplemented with 20% FBS. After 24h of incubation, wiped the upper chamber carefully to removed non-migrated cells, followed by fixing and staining the cells that traversed the membrane using 0.1% crystal violet. After PBS washing for several times, cell numbers in five random fields were counted in each sample. For tube formation assay, 2×10^5 EPCs were incubated in a 24-well plate,

which was pre-cooled and replenished with 250 μl of Matrigel (Corning, NY, USA). Cells were then incubated with 500µL AB-media, cells incubated with complete media as vehicle. After 6 hours, tubes linked by endothelial cells were observed and quantified as previously described.[49]

In vitro assay for osteogenic differentiation of MSCs

For osteogenic differentiation, 1×10^6 MSCs were incubated in 24-well plates and induced with AB-contained osteogenic medium for 14 days and 21 days, respectively. After 14 and 21 days of induction, total RNAs and proteins were harvested for RT-qPCR and western blot. In addition, ALP activity was assessed using ALP stain kit (Abcam) after 14 days induction. For ARS staining, cells induced for 14 days were fixed and stained with 2% Alizarin red-S (Sigma). The calcium deposits were observed and quantified under microscope.

Preparation of decalcified bone matrix (DBM) loaded with ABs

DBMs were detached from limb bones of cattle and treated as previously described. [50] After that, DBMs were divided into 2.5 mm × 2.5 mm blocks, and the blocks later were soaked with 75% ethanol for 3 hours and rinsed with PBS. Then ABs were re-suspended in fibronectin gel (Corning) at a final concentration of 1µg /uL. Subsequently, the gel was added onto the DBMs and incubated at 37 °C for 12 hours. Finally, the DBMs was dried and frozen at -70 for further experiments.

Animal experiments

We obtained *Pdgfb*$^{f/f}$ and *Tnfrsf11a*$^{f/f}$ mice from Jackson Laboratory, *TRAP-cre* mouse strain source was previously described.[51] We crossed *TRAP-cre* mice with *Pdgfb*$^{f/f}$ or *Tnfrsf11a*$^{f/f}$ mice to obtain *TRAP-cre*;*Pdgfb*$^{f/f}$ mice and *TRAP-cre*;*Tnfrsf11a*$^{f/f}$ mice. To observe the osteogenic potency of DBM loaded with ABs, we established cranial defect mice model. Briefly, C57BL/6 mice aged 4-6 weeks was anesthetized. A 2 cm incision was made in the center of the mouse head to expose the cranium, and a dental drill was used to make two 2.5 mm defects on both sides of the cranium. After that, DBM loaded with ABs were embedded in the bone defects. 32 mice were randomly divided into four groups according to the distinctive types of ABs loaded by DBM. Cranium samples were taken two weeks and four weeks after surgery for further investigation. All animal breeding and experimental procedures have been approved by the Institutional Animal Care and Use Committee of Johns Hopkins University School of Medicine.

µCT analysis

Bruker MicroCT Skyscan 1272 system (Kontich, Belgium) was used to capture the images of the whole mouse calvarial bone with an isotropic voxel size of 10.0 µm. Scanning was done using a 60 kV X-ray tube with an X-ray intensity of 166 µA at an exposure time of 1700

ms. 3D reconstruction of the CT images was performed for the region of grafting containing the DBM or drilling sites. Reconstruction was performed using software Nrecon (Kontich, Belgium). 3D and 2D analysis were performed using software CT Analyser (Ver. 1.15.4.0, Kontich, Belgium). 3D images were acquired from contoured 2D images by methods based on distance transformation of the gray scale original images (Ver. 3.0.0, CTvox, Kontich, Belgium).

Immunofluorescent Staining for $CD31^{hi}Emcn^{hi}$ Vessels

To investigate the expression of CD31 and endomucin of newly formed tissue, we performed immunofluorescent staining on the paraffin sections. In short, individual mouse-anti-CD31 (1:200, Bioss) and rabbit-anti-endomucin (1:200, Bioss) were used to incubate sections at 4°C overnight. Then sections were incubated with corresponding fluorescence-labeled secondary antibody at room temperature in dark for 1h. Images of sections were captured using A Leica TCS SP8.

Histological and immunohistochemistry evaluation

Mouse cranial bones were collected at 2 weeks (for angiogenesis evaluation) or 4 weeks (for osteogenesis evaluation) after surgery, respectively. The process of making cranial bones into bone sections has been described in our previous study.[49] After fixation, decalcification and paraffin embedding, the histological sections were cut into 1 μm of thickness and were prepared for subsequent H&E staining. For IHC assessment, the expressions of CD31, OCN and p-SMAD2/3 were detected according to the following procedure. Sections were incubated with individual rabbit-anti-CD31, rabbit-anti-OCN and rabbit-anti-p-SMAD2/3 at 37℃ for 2 hours, followed by incubation with Biotinylated secondary antibody (1% BSA-PBS dilution). After DBA Chromogenic Kit (Ybscience) were used for chromogenic reaction, the stained sections were washed and observed using light microscopy. German semi-quantitative method was used to evaluate IHC score, the specific scoring rules have been explained in previous studies.[49]

Statistical analysis

The data are presented as means ± standard deviation. Student's t-test was employed to compare two individual groups. One-way ANOVA followed by Student-Newman-Keuls post hoc tests were employed to analyze multiple sets of data. All experiments with data were repeated at least three times. $p < 0.05$ was regarded as statistically significant, indicated as $*p < 0.05$, $**p < 0.01$.

Acknowledgments

This work was funded by the grant from the National Natural Science Foundation of China (81802166), the State Key Program of National Natural Science of China (81930067) and

first class General Financial Grant from the China Postdoctoral Science Foundation (2017M613315).

Conflict of interest

The authors report no conflict of interest.

Figure legends

Fig. 1 Isolation and characterization of ABs from BMMs, pOCs and mOCs

(a) Graphical illustration of ABs isolation.

(b) Images of TRAP staining and immunofluorescent stain (IF) of cytoskeleton of BMMs, pOCs and mOCs. Cell nuclei stained with DAPI were shown in blue and vinculin were shown in red. Scale bars represent 200 µm.

(c) The proportion of TRAP-positive cells and multinucleated TRAP-positive cells was quantified in each well (96 well plate), n = 5.

(d) Heat map showed the expression profile of RANKL-dependent specific genes of osteoclastogenesis from 0h (BMMs) to 96h (mOCs).

(e) Representative images of ABs stained with Annexin-V/FITC. Bar represents 3 µm.

(f) Forward/side scatter (FSC/SSC) analysis and Annexin-V/PI analysis of viable cells, apoptotic cells and ABs.

The data in the figures represent the averages ± SD. Statistically significant differences between the treatment and control groups are indicated as * ($p < 0.05$) or ** ($p < 0.01$).

Fig. 2 Proteomic profiling of ABs from BMMs, pOCs and mOCs

(a) Hierarchical clustering heat map showed the expression profile of 4,306 proteins identified in ABs.

(b) VENN diagrams comparing total proteome identified in distinctive ABs.

(c) Subcellular localization analysis showed the distribution of all significantly expressed proteins (DEPs) in cell structure. DEPs were mainly located in cytoplasm, nucleus and mitochondria.

(d) Volcano plots showed DEPs in three comparison groups. Red dots represent differentially upregulated proteins (DUPs, FC > 2.0) and blue dots represent downregulated proteins (DDPs, FC < 0.5).

Fig. 3 Resemblances of proteomic signatures of ABs with parental cells indicates functional similarities.

(a) VENN analysis of three comparison groups screened out the proteomic signatures of different ABs. The threshold of FC is > 2.0 or < 0.5. (G1, pOC-ABs *versus* BMM-ABs; G2, mOC-ABs *versus* BMM-ABs; G3, mOC-ABs *versus* pOC-ABs).

(b) Proteomic signatures of ABs from BMMs, pOCs and mOCs were shown by heat map. Among them, PDGF-BB and RANK were proteomic signatures of pOC-ABs and mOC-ABs respectively.

(c) Principal-components analysis (PCA) of protein signatures expressed in three ABs. The elliptical solid line indicates 95% confidence intervals.

(d) VENN diagrams comparing candidate proteomic signatures of ABs with the gene signatures of parental and non-parental cells.

GSEA plots showed the relevance of whole proteomes of (e) pOC-ABs and (f) mOC-ABs with gene expression profiles in parental cells.

GO analysis revealed that (g) DUPs in pOC-ABs were enriched in angiogenesis-associated BP terms whereas (h) mOC-ABs DUPs were more enriched in osteogenesis BP terms.

GSEA analysis showed that (i) enriched proteins in pOC-ABs were more closely associated with angiogenesis-related biological processes, whereas (j) enriched proteins in mOC-ABs were associated with osteogenic activities.

Fig. 4 pOC-ABs and mOC-ABs inherited distinct biological functions from the parental cells.

(a) Schematic diagram of ABs engulfment investigation.

Representative images of confocal microscopy analysis of cell tracker CM-DiI labeled (b) EPCs or (c) MSCs (red) incubated with Annexin-V/FITC labeled ABs (green). The merged panel was shown on the left. White arrows represents ABs were engulfed by recipient cells. Bar represents 10 μm.

(d) EPCs viability was assessed at 1, 3 and 7 days after treated with different ABs. Vehicle represents EPCs cultured with complete media.

(e) Tube formation assay of EPCs cultured with ABs for 6 hours. Representative images (left panel) and quantification of calculative tube length (right panel) were shown. Bar represents 100 μm, n = 5.

(f) Protein expressions of phosphorylated PI3K and AKT in EPCs were detected by western blot.

(g) RT-qPCR analysis of *Pecam1*, *ANG-1* and *Kdr* expression in EPCs treated with distinct AB-media.

(h) Representative ALP staining images and quantification of ALP activity. Vehicle represents MSCs cultured with complete media, n = 5.

(i) Representative Alizarin staining images and quantitative analysis of calcium deposit of MSCs. Vehicle represents MSCs cultured with complete media, n = 5.

(j) RT-qPCR analysis of *Alpl*, *Osx*, *Runx2* and *Col1a1* expression of MSCs cultured with different AB-media.

(k) Western blots of osteogenic markers Collagen Ⅰ and RUNX2 in MSCs cultured with ABs.

The data in the figures represent the averages ± SD. Statistically significant differences between the treatment and control groups are indicated as * ($p < 0.05$) or ** ($p < 0.01$).

Fig. 5 pOC-ABs promote angiogenesis via delivering PDGF-BB to recipient EPCs.

(a) Immunohistochemistry (IHC) of CD31 in mice grafted with AB-DBM for 2 weeks. Red arrows indicate CD31 positive new vessels formed in defect repair area. Bar represent 1 mm (upper) and 100 μm (lower).

(b) Semi-quantitative analysis showed the IHC score of CD31 in mice grafted with AB-DBM, n = 8.

(c) Representative flowcytometry analysis with quantitive percentage of CD31^{hi}Emcn^{hi} cells in total cells digested from defect repair area (DRA), n = 5.

(d) Immunostaining analysis of CD31 and Emcn with quantitative analysis of relative immunostaining intensities in DRA. Bar represent 40 μm, n = 5.

(e) PDGF-BB expression in different ABs by western blots. As shown, PDGF-BB is one of the proteomic signatures of pOC-ABs.

(f) PDGF-BB expression in pOC-ABs extracted from pOCs of *TRAP-cre;Pdgfb*$^{f/f}$ mice by western blots.

(g) IHC and semi-quantitative analysis of CD31 expression in mice grafted with DBM pre-incubated with *TRAP-cre;Pdgfb*$^{f/f}$ pOC-ABs or *Pdgfb*$^{f/f}$ pOC-ABs. Bar represent 1 mm of overview and 100 μm of center region, n = 8.

(h) Representative flowcytometry analysis with percentage of CD31^{hi}Emcn^{hi} cells in total cells digested from DRA, n = 5.

(i) Representative images of immunostaining of CD31 and Emcn with quantification of relative CD31 and Emcn immunostaining intensities in DRA. Bar represent 20 μm, n = 5.

The data in the figures represent the averages ± SD. Statistically significant differences between the treatment and control groups are indicated as * ($p < 0.05$) or ** ($p < 0.01$).

Fig. 6 mOC-ABs promote osteogenesis through RANKL reverse signaling.

(a) Representative micro-CT images of cranial bone, 4 weeks after AB-DBM implantation.

(b) Quantification of bone histomorphometry parameters (bone formation ratio, BV/TV, BMD) were measured based on micro-CT images, n = 8.

(c) Representative histological images for Masson staining, and IHC of osteocalcin (OCN) and p-SMAD2/3 from mice cranial bone sections (left). Quantification of bone formation ratio and semi-quantitative analysis of OCN and p-SMAD2/3 in mice grafted with AB-DBM (right). Scale bars, 300 μm for Masson staining and 70 μm for IHC staining, n = 8.

(d) Western blot analysis of RANK levels in different ABs. As shown, RANK is one of the proteomic signatures of mOC-ABs.

(e) Representative micro-CT images of cranial bone, treated with *TRAP-cre;Tnfrsf11a*$^{f/f}$ mOC-ABs for 4 weeks.

(f) Quantification of the amount of new bone formation and BMD of total DRA of mice treated with *TRAP-cre;Tnfrsf11a*$^{f/f}$ mOC-ABs were measured based on micro-CT images, n = 8.

(g) Representative histological images for Masson staining, and IHC of OCN and p-SMAD2/3 in indicated groups (left). Quantification of semi-quantitative analysis of OCN and p-SMAD2/3 and bone formation ratio in indicated groups (right). Scale bars, 300 μm for Masson staining and 70 μm for IHC staining, n = 8.

The data in the figures represent the averages ± SD. Statistically significant differences between the treatment and control groups are indicated as * ($p < 0.05$) or ** ($p < 0.01$).

Figure 7. Schematic diagram showing the role of osteoclast-derived ABs in bridging bone resorption and formation in bone remodeling.

①A fraction of pOCs undergo apoptosis and generate pOC-ABs.

②pOC-ABs induce formation of CD31^{hi}Emcn^{hi} vessels via delivering PDGF-BB to recipient EPCs.

③pOCs differentiate into mOC upon stimulation of RANKL and M-CSF.

④mOCs undergo apoptosis and generate mOC-ABs at the end of bone resorption phase.

⑤mOC-ABs promote osteogenesis through RANKL reverse signaling. Besides, CD31^{hi}Emcn^{hi} vessels induced by pOC-ABs improve local nutrients and metabolic wastes transport to further maintain the integrity of bone remodeling.

CC_200908G700

ORIGINALITY REPORT

14%

SIMILARITY INDEX

PRIMARY SOURCES

1	Qinyu Ma, Mengmeng Liang, Nathachit Limjunyawong, Yang Dan, Junchao Xing, Jianmei Li, Jianzhong Xu, Ce Dou. "Osteoclast-derived apoptotic bodies show extended biological effects of parental cell in promoting bone defect healing", Theranostics, 2020<br>Crossref	334 words — 5%
2	link.springer.com<br>Internet	120 words — 2%
3	Qinyu Ma, Mengmeng Liang, Yutong Wu, Ning Ding, Lianli Duan, Tao Yu, Yun Bai, Fei Kang, Shiwu Dong, Jianzhong Xu, Ce Dou. "Mature osteoclast–derived apoptotic bodies promote osteogenic differentiation via RANKL-mediated reverse signaling", Journal of Biological Chemistry, 2019<br>Crossref	53 words — 1%
4	www.oncotarget.com<br>Internet	48 words — 1%
5	tessera.spandidos-publications.com<br>Internet	39 words — 1%
6	www.ncbi.nlm.nih.gov<br>Internet	36 words — < 1%
7	Keping Ye, Jie Haung, Cong Zhou, Guanghong Zhou. " TMT-Based Quantitative Proteomic Analysis of Intestinal Organoids Infected by with Different Virulence ", Cold Spring Harbor Laboratory, 2020<br>Crossref	29 words — < 1%

8 www.hindawi.com
Internet
28 words — < 1%

9 Ce Dou, Ning Ding, Fei Luo, Tianyong Hou, Zhen Cao, Yun Bai, Chuan Liu, Jianzhong Xu, Shiwu Dong. "Graphene-Based MicroRNA Transfection Blocks Preosteoclast Fusion to Increase Bone Formation and Vascularization", Advanced Science, 2018
Crossref
27 words — < 1%

10 www.nature.com
Internet
25 words — < 1%

11 www.spandidos-publications.com
Internet
22 words — < 1%

12 Zengwen Huang, Juan Zhang, Yaling Gu, Guosheng Xin, Zhengyun Cai, Xianfeng Yin, Xiaofang Feng, Tong Mu, Chaoyun Yang. "Proteomics Analysis and Identification of Critical Proteins and Network Interactions That Regulate the Specific Deposition of IMF of Jingyuan Chicken", Research Square, 2020
Crossref Posted Content
20 words — < 1%

13 Ning Ding, Chuan Liu, Li Yao, Yun Bai et al. "Alendronate induces osteoclast precursor apoptosis via peroxisomal dysfunction mediated ER stress", Journal of Cellular Physiology, 2018
Crossref
19 words — < 1%

14 Weiping Su, Guanqiao Liu, Xiaonan Liu, Yangying Zhou et al. "Angiogenesis stimulated by elevated PDGF-BB in subchondral bone contributes to osteoarthritis development", JCI Insight, 2020
Crossref
16 words — < 1%

15 ajpgi.physiology.org
Internet
16 words — < 1%

16 e-sciencecentral.org
Internet
15 words — < 1%

17 Chuan Liu, Zhen Cao, Wen Zhang, Jennifer Tickner, Heng Qiu, Chao Wang, Kai Chen, Ziyi Wang, Renxiang Tan, Shiwu Dong, Jiake Xu. "Lumichrome inhibits osteoclastogenesis and bone resorption through suppressing RANKL-induced NFAT activation and calcium signaling", Journal of Cellular Physiology, 2018 — 15 words — < 1%
Crossref

18 Zhou, Yu, Xing-Hui Li, Cai-Cai Zhang, Ming-Jie Wang, Wen-Long Xue, Dong-Dong Wu, Fen-Fen Ma, Wen-Wen Li, Bei-Bei Tao, and Yi-Chun Zhu. "Hydrogen sulfide promotes angiogenesis by down-regulating miR-640 via the VEGFR2/mTOR pathway", AJP Cell Physiology, 2015. — 14 words — < 1%
Crossref

19 bmcplantbiol.biomedcentral.com — 14 words — < 1%
Internet

20 Norihiro Ishida-Kitagawa, Kunitaro Tanaka, Xilinqiqige Bao, Takanori Kimura et al. "Siglec-15 Protein Regulates Formation of Functional Osteoclasts in Concert with DNAX-activating Protein of 12 kDa (DAP12)", Journal of Biological Chemistry, 2012 — 12 words — < 1%
Crossref

21 Ce Dou, Ning Ding, Fei Luo, Tianyong Hou, Zhen Cao, Yun Bai, Chuan Liu, Jianzhong Xu, Shiwu Dong. "Graphene-Based MicroRNA Transfection Blocks Preosteoclast Fusion to Increase Bone Formation and Vascularization", Advanced Science, 2017 — 11 words — < 1%
Crossref

22 cmbl.biomedcentral.com — 10 words — < 1%
Internet

23 www.tandfonline.com — 10 words — < 1%
Internet

24 pubs.rsc.org — 9 words — < 1%
Internet

25 Yukiko Komano. Arthritis Research & Therapy, 2006
Crossref
9 words — < 1%

26 authors.library.caltech.edu
Internet
9 words — < 1%

27 Yanai Peled, Jeana Drake, Assaf Malik, Ricardo Almuly, Maya Lalzar, David Morgenstern, Tali Mass. " Optimization of skeletal protein preparation for LC-MS/MS sequencing yields additional coral skeletal proteins in ", Cold Spring Harbor Laboratory, 2020
Crossref Posted Content
9 words — < 1%

28 www.researchsquare.com
Internet
9 words — < 1%

29 theses.gla.ac.uk
Internet
8 words — < 1%

30 Corinne Decker, Pamela Hesker, Kaihua Zhang, Roberta Faccio. "Targeted Inhibition of Phospholipase C γ2 Adaptor Function Blocks Osteoclastogenesis and Protects from Pathological Osteolysis", Journal of Biological Chemistry, 2013
Crossref
8 words — < 1%

31 curis.ku.dk
Internet
8 words — < 1%

32 Dou, Ce, Zhen Cao, Ning Ding, Tianyong Hou, Fei Luo, Fei Kang, Xiaochao Yang, Hong Jiang, Zhao Xie, Min Hu, Jianzhong Xu, and Shiwu Dong. "Cordycepin Prevents Bone Loss through Inhibiting Osteoclastogenesis by Scavenging ROS Generation", Nutrients, 2016.
Crossref
7 words — < 1%

33 mdpi.com
Internet
6 words — < 1%

34 Qiang Guo, Zhen Cao, Bo Wu, Fangxiao Chen et al. "Modulating calcium-mediated NFATc1 and mitogen-
6 words — < 1%

activated protein kinase deactivation underlies the inhibitory effects of kavain on osteoclastogenesis and bone resorption", Journal of Cellular Physiology, 2019
Crossref

---

35 Xiao-Qing Ding, Zhe-Ying Wang, Di Xia, Rui-Xian Wang, Xiao-Rong Pan, Jian-Hua Tong. "Proteomic Profiling of Serum Exosomes From Patients With Metastatic Gastric Cancer", Frontiers in Oncology, 2020 — 6 words — < 1%
Crossref

---

36 Feng Xiong, Zainen Qin, Haimin Chen, Qiumei Lan, Zetao Wang, Nihan Lan, Yuan Yang, Li Zheng, Jinmin Zhao, Dan Kai. "pH-responsive and hyaluronic acid-functionalized metal-organic frameworks for therapy of osteoarthritis", Research Square, 2020 — 6 words — < 1%
Crossref Posted Content

---

37 www.physiology.org — 6 words — < 1%
Internet

---

38 Yuko Tanabe, Yusuke Naito, Cristina Vasuta, Alfred Kihoon Lee, Youssouf Soumounou, Michael W. Linhoff, Hideto Takahashi. "IgSF21 promotes differentiation of inhibitory synapses via binding to neurexin2α", Nature Communications, 2017 — 6 words — < 1%
Crossref

---

39 Samuel C. Ramage, Nicole H. Urban, William A. Jiranek, Aparna Maiti, Matthew J. Beckman. "Expression of RANKL in Osteolytic Membranes", The Journal of Bone & Joint Surgery, 2007 — 6 words — < 1%
Crossref

EXCLUDE QUOTES	OFF	EXCLUDE MATCHES	OFF
EXCLUDE BIBLIOGRAPHY	ON		
