# Supplementary material for: Osteoclast-derived apoptotic bodies couple bone resorption and formation in bone remodeling
Source: Bone Res. 2021 Jan 11;9:5. doi: 10.1038/s41413-020-00121-1 (PMC7801485; doi:10.1038/s41413-020-00121-1)
Supplement: Supplementary file 2 — Osteoclast-derived apoptotic bodies bridge bone resorption and formation in bone remodeling [file 41413_2020_121_MOESM2_ESM.docx]

**Osteoclast-derived apoptotic bodies bridge bone resorption and formation in bone remodeling**

Qinyu Ma ^1#^, Mengmeng Liang ^2#^, Yutong Wu ^1^, Fei Luo ^1^, Zaisong Ma ^3^, Shiwu Dong ^2,4*^, Jianzhong Xu ^1*^, and Ce Dou ^1,5*^

^1^ Department of Orthopedics, Southwest Hospital, Third Military Medical University, Chongqing 400038, China.

^2^ Department of Biomedical Materials Science, Third Military Medical University, Chongqing 400038, China.

^3^ Department of Orthopedics, General Hospital of Xinjiang Military Command, Urumqi, Xinjiang, 830000, China.

^4^ State Key Laboratory of Trauma, Burns and Combined Injury, Third Military Medical University, Chongqing 400038, China.

^5^ Department of Orthopedic Surgery, Johns Hopkins University School of Medicine, Baltimore, Maryland 21205, USA.

^#^ These authors contribute equally to this article

**Corresponding author:**

Shiwu Dong, Department of Biomedical Materials Science, Third Military Medical University, Chongqing 400038, China. Tel: +86 13883788486 Email: dongshiwu@tmmu.edu.cn

Jianzhong Xu, Department of Orthopedics, Southwest Hospital, Third Military Medical University, Gaotanyan Street No.30, Chongqing 400038, China. Tel: +86 13883318800 Email: xujianzhong1962@163.com

Ce Dou, Department of Orthopedics, Southwest Hospital, Third Military Medical University, Gaotanyan Street No.30, Chongqing 400038, China. Tel: +86 13883443401 Email: [lance.douce@gmail.com](mailto:lance.douce@gmail.com)

**Keywords:** Apoptotic body, Intercellular communication, Vesicle bioinformatics

**Supplementary methods and materials**

Western blot analysis

Total proteins from different groups were lysed in radio immune precipitation assay buffer (Beyotime Biotechnology, Nantong, Jiangsu, China) containing a protease inhibitor cocktail and incubated on ice for 30 min. After centrifugation at 13000 g, the supernatant was carefully removed and transferred into a new microfuge tube. The concentration of proteins was detected using the bicinchoninic acid protein assay kit (Beyotime Biotechnology, China). Each sample (50 μg) was diluted in loading buffer and subjected to a standard SDS-PAGE followed by transferred onto PVDF membranes (Immobilon^TM^-PSQ Membranes, Sigma-Aldrich, Saint Louis, MO, USA). After blocking in 5% skim milk, proteins were detected using the following antibodies: RUNX2 (bs-1134R) at a 1:1000 dilution, Collagen I (bs-10423R) at a 1:1000 dilution, PDGF-BB (bs-1316P) at a 1:1000 dilution, RANK (bs-7343R) at a 1:1000 dilution, PDGF-BB (ab23914) at a 1:1000 dilution, CASP9 (bs-0049R) at a 1:1000 dilution, PARP (bs-2138R) at a 1:1000 dilution, PI3K (bs-10657R) at a 1:1000 dilution, p-PI3K (bs-6417R) at a 1:1000 dilution, AKT (bs-0115M) at a 1:1000 dilution, p-AKT (bs-2720R) at a 1:1000 dilution and GAPDH (bs-0755R) at a 1:2000 dilution. Corresponding secondary antibodies against primary antibodies were used by an hour of incubation (1:2000). Blots against GAPDH served as loading control. Chemiluminescent signals were detected using Immun-Star HRP (BioRad).

RT-qPCR analysis

Total RNAs from different groups were extracted using Trizol reagent (Life Technologies, NY, USA) according to the manufacturer’s instructions. Concentrations of the RNA samples were measured using the ultra-low volume spectrometer (BioDrop µLite, Cambridge, England). For real-time PCR of mRNA, the cDNA was synthesized using PrimeScript^TM^RT reagent kit (Takara, Nojihigashi, Japan) according to the manufacturer’s instructions. The real-time PCR was performed using SYBR Green Supermix (Bio-Rad, Hercules, CA, USA) and specific primers listed in supplementary Table 1. Real-time PCR was detected on a CFX96™ Real-Time PCR System instrument (Bio-Rad).

μCT analysis

Bruker MicroCT Skyscan 1272 system (Kontich, Belgium) was used to capture the images of the whole mouse calvarial bone with an isotropic voxel size of 10.0 μm. Scanning was done using a 60 kV X-ray tube with an X-ray intensity of 166 μA at an exposure time of 1700 ms. 3D reconstruction of the CT images was performed for the region of grafting containing the DBM or drilling sites. Reconstruction was performed using software Nrecon (Kontich, Belgium). 3D and 2D analysis were performed using software CT Analyser (Ver. 1.15.4.0, Kontich, Belgium). 3D images were acquired from contoured 2D images by methods based on distance transformation of the gray scale original images (Ver. 3.0.0, CTvox, Kontich, Belgium).

ABs engulfment assay and MFI analysis

ABs were stained with Annexin V-FITC (50mg/ml) for 20 min at room temperature and EPCs were stained with cell tracker red for 20 min at room temperature. Then, ABs stained with Annexin-V were pre-incubated with EPCs stained with cell tracker red for 24h. After full co-culturing time according to group settings, washed off the ABs that have not been engulfed by EPC then observed using confocal microscopy to validate the engulfment of ABs. The FITC fluorescence of EPCs was detected by flowcytometry, which counted at last 20000 events. MFI was calculated and generated from Flowjo-V10 software.

**Supplementary Figures**


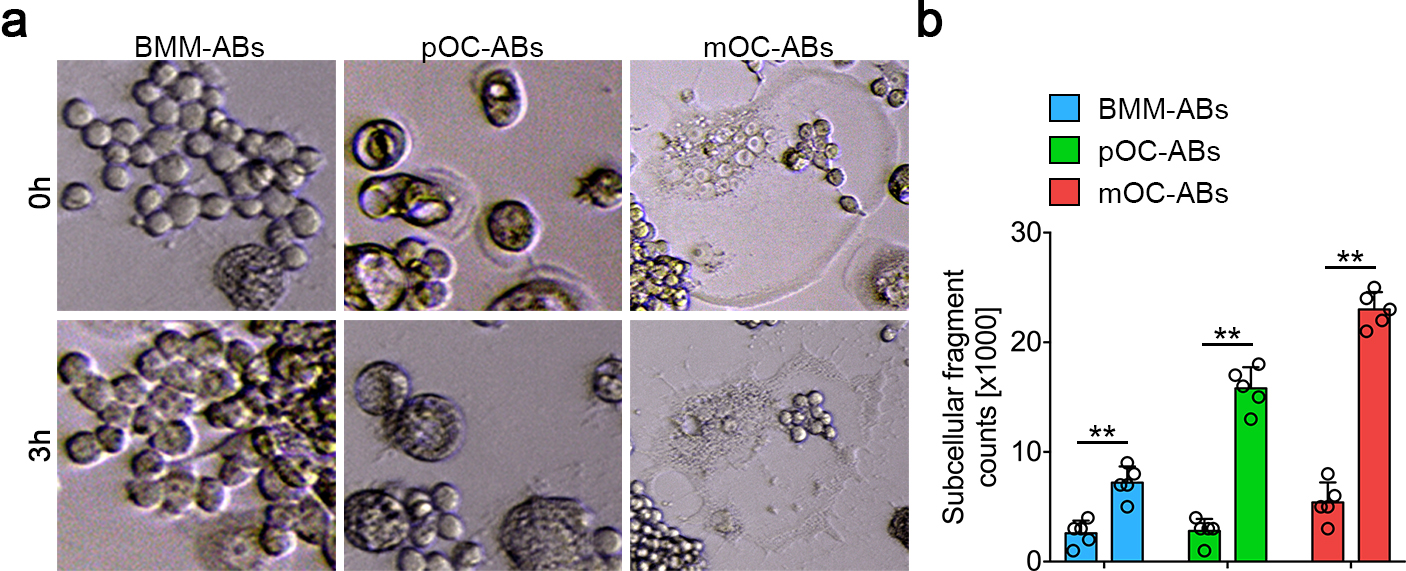


**Fig. S1 Generation of BMM-ABs, pOC-ABs and mOC-ABs.**

(a) Apoptosis of BMMs, pOCs and mOCs were induced with STS (5 μM) and observed using light microscopy after 3 hours induction.

(b) Quantification of subcellular fragment counts. The data in the figures represent the averages ± SD.

Significant differences are indicated as * (*p* < 0.05) or ** (*p* < 0.01) paired using Student’s t test unless otherwise specified.


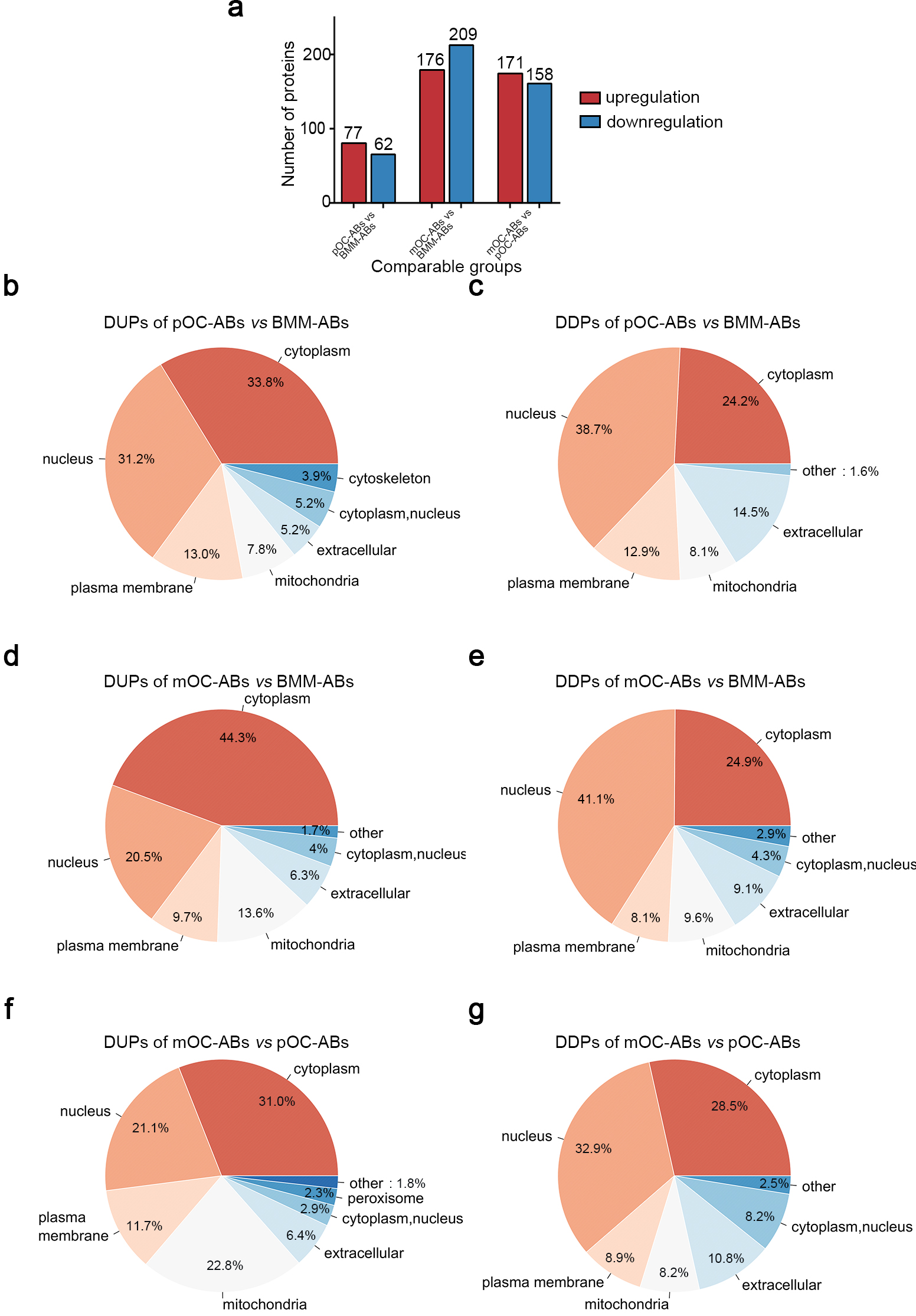


**Fig. S2 Proteomic profiling of BMM-ABs, pOC-ABs and mOC-ABs.**

(a) The whole distribution of all differential expressed proteins (DEPs) in three comparison groups was shown.

Subcellular localization ratio of DUPs in (b) pOC-ABs *vs* BMM-ABs (d) mOC-ABs *vs* BMM-ABs (f) mOC-ABs *vs* pOC-ABs, and DEPs in (c) pOC-ABs *vs* BMM-ABs (e) mOC-ABs *vs* BMM-ABs (g) mOC-ABs *vs* pOC-ABs was shown.


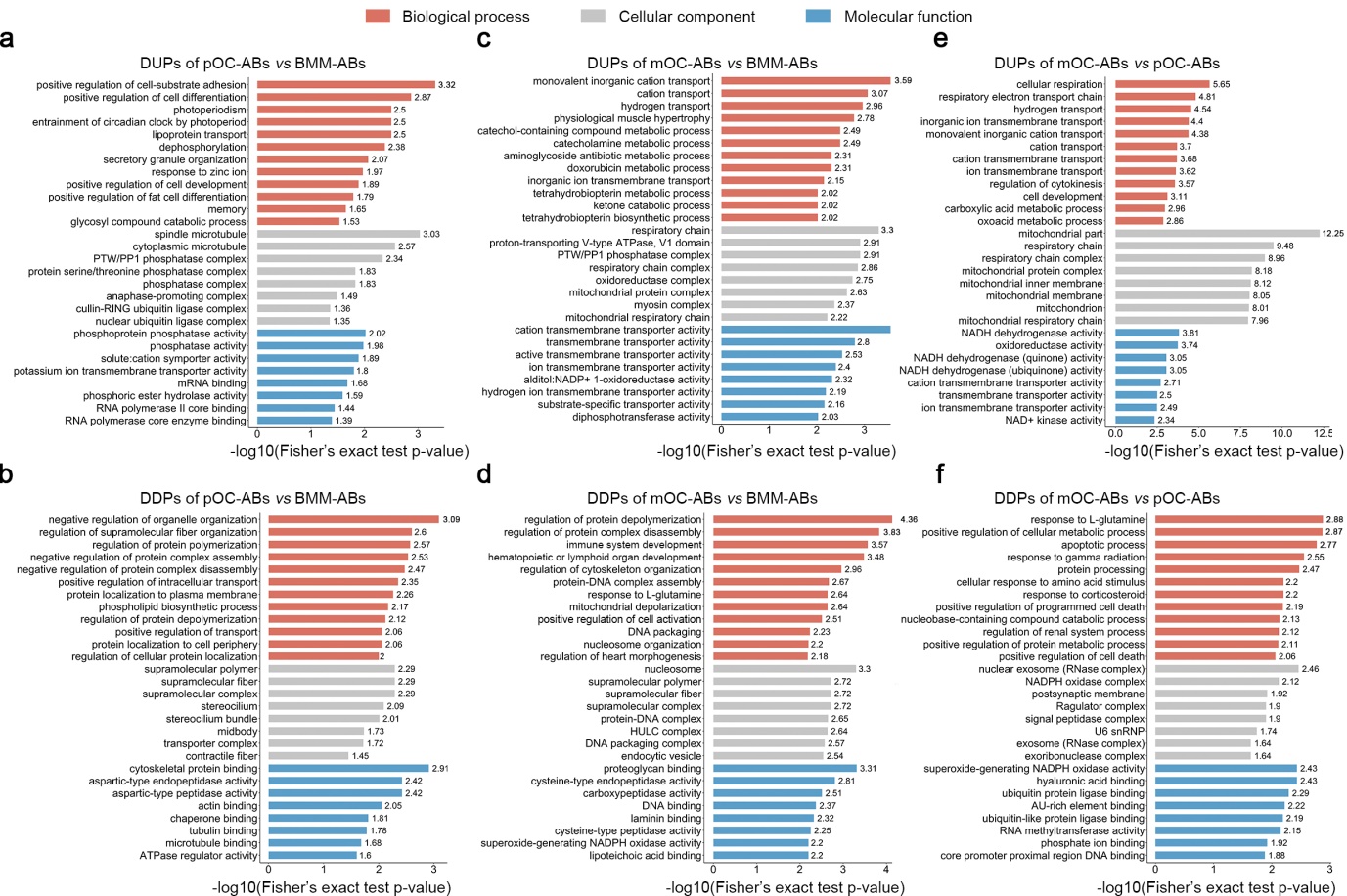


**Fig. S3 GO enrichment analysis of ABs.**

GO enrichment analysis was performed and GO terms ranked by enrichment score of DUPs in (a) pOC-ABs *vs* BMM-ABs (b) mOC-ABs *vs* BMM-ABs (c) mOC-ABs *vs* pOC-ABs, and DEPs in (d) pOC-ABs *vs* BMM-ABs (e) mOC-ABs *vs* BMM-ABs (f) mOC-ABs *vs* pOC-ABs were shown.


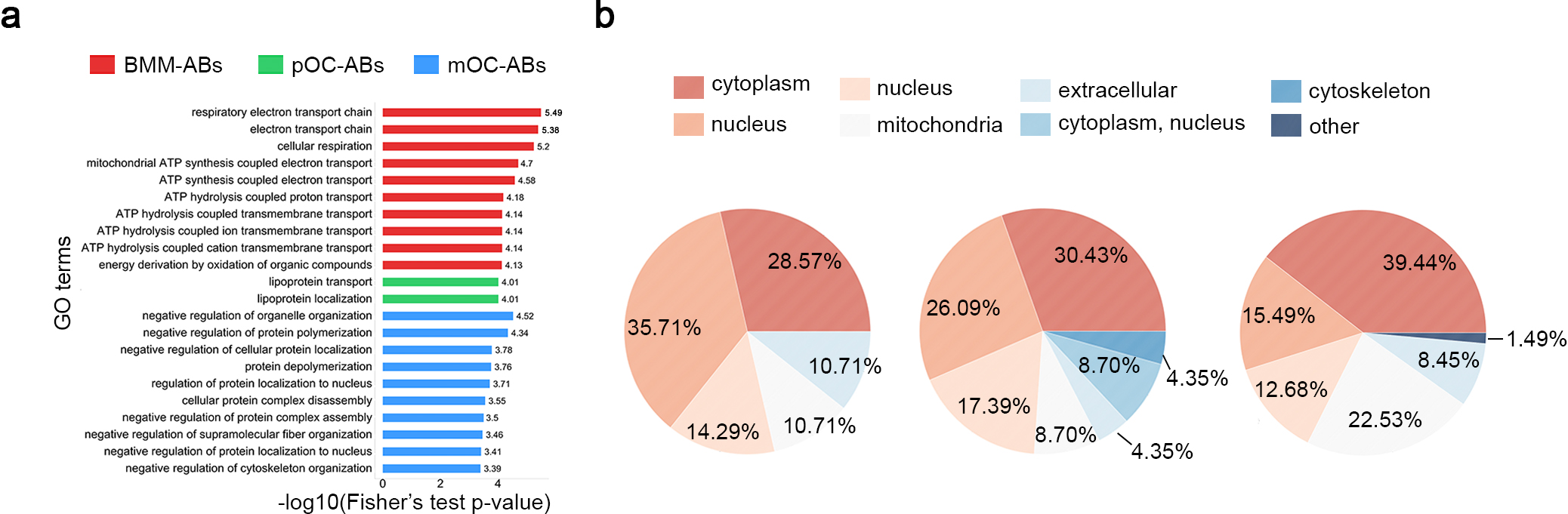


**Fig. S4** **GO enrichment analysis and subcellular localization of AB signatures.**

(a) GO enrichment analysis and (b) subcellular structure localization of protein signatures of three ABs were shown.


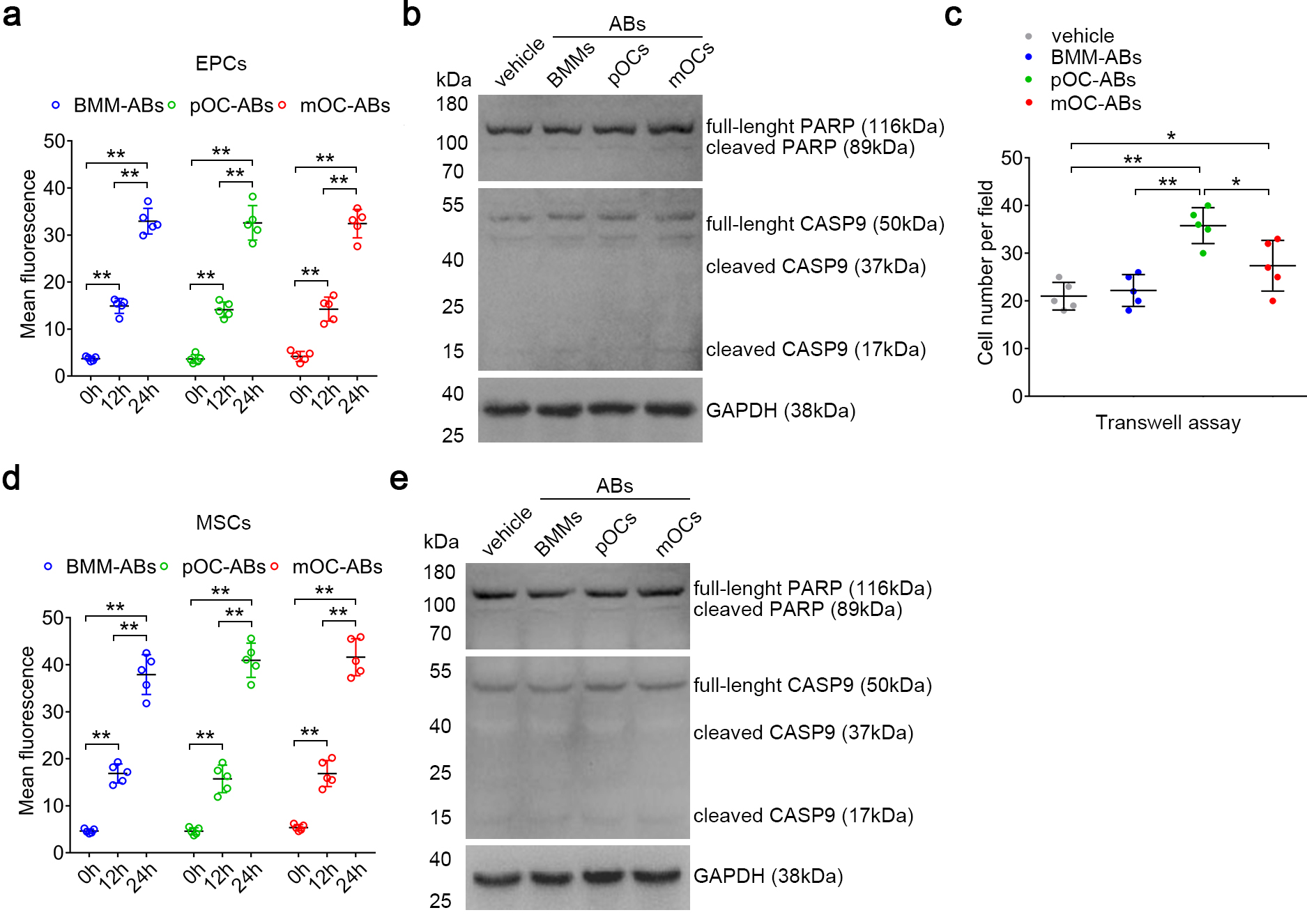


**Fig. S5** **pOC-ABs and mOC-ABs inherited specific biological functions from the parental cells.**

(a) Quantification of mean fluorescence intensity of engulfed ABs by EPCs after co-incubation. n = 5.

(b) WB analysis of EPCs cultured with three ABs for 24h. The absence of cleavage and activation of the apoptosis-related proteins PARP and CASP9 indicates a lack of activation of apoptotic pathways.

(c) Transwell assay of EPCs after cultured with three ABs for 24h. Vehicle represents EPCs without any treatment.

(d) Quantification of mean fluorescence intensity of engulfed ABs by MSCs after co-incubation. n = 5.

(e) WB analysis of MSCs co-incubated with three ABs for 24h. The absence of cleavage and activation of the apoptosis-related proteins PARP and CASP9 indicates a lack of activation of apoptotic pathways.

The data in the figures represent the averages ± SD. Significant differences are indicated as * (*p* < 0.05) or ** (*p* < 0.01) paired using Student’s t test unless otherwise specified.


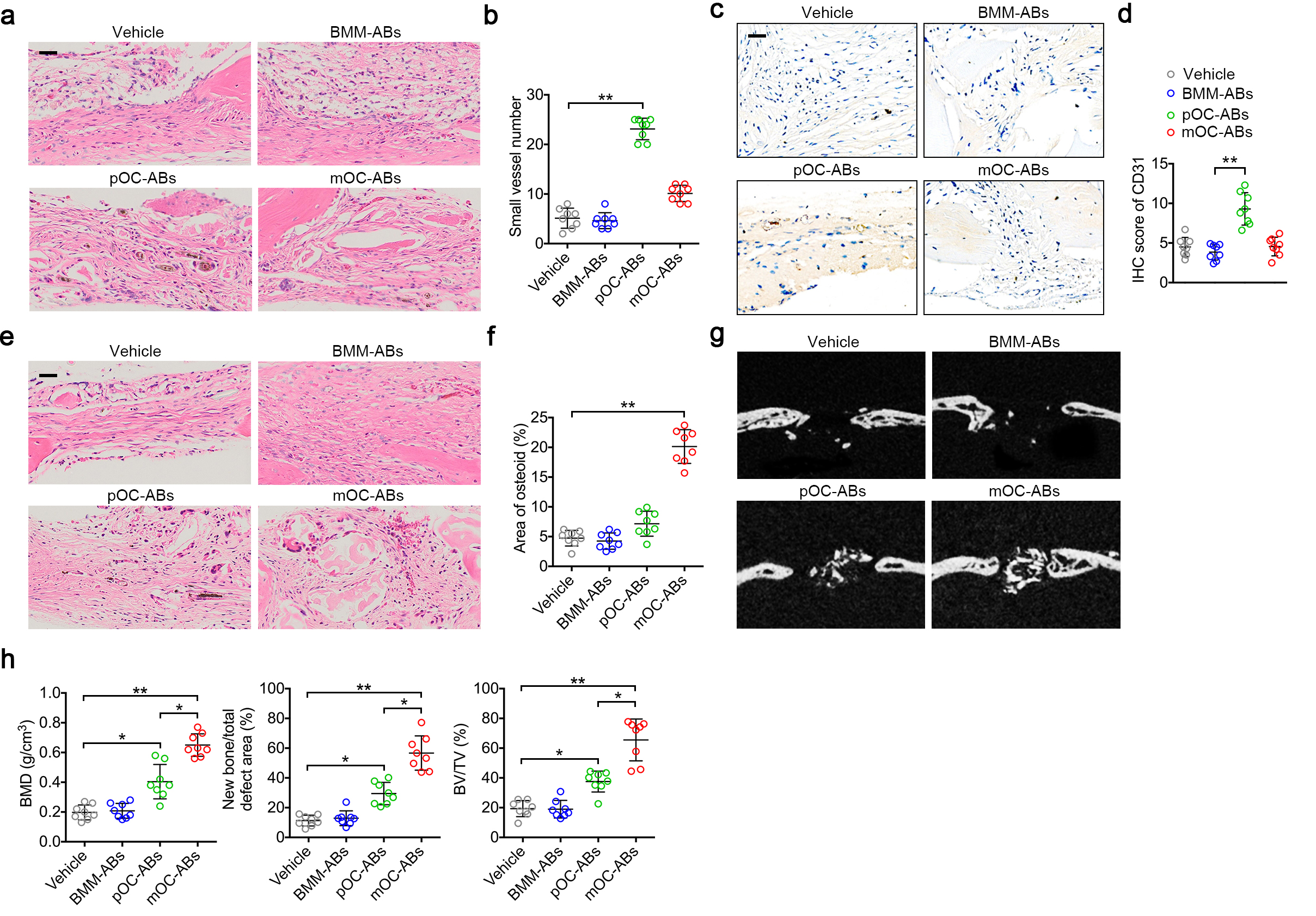


**Fig. S6** **pOC-ABs promote angiogenesis and mOC-ABs promote osteogenesis *in vivo*.**

(a) Representative images of H&E staining images and (b) quantitative analysis of small vessel number of mice treated with three ABs for two weeks. n = 8. Bar represent 100 μm.

(c) IHC of CD31 in female mice grafted with AB-DBM for 4 weeks. Bar represent 100 μm.

(d) Semi-quantitative analysis showed the IHC score of CD31 in female mice grafted with AB-DBM, n = 8.

(e) Representative images of H&E staining images and (f) quantitative analysis of osteoid area (%) of mice treated with three ABs for four weeks. n = 8. Bar represent 100 μm.

(g) Representative coronal micro-CT images of female mice grafted with AB-DBM for 4 weeks.

(h) Quantitative micro-CT analysis showed the amount of bone formation, bone volume density (BV/TV), and bone mineral density (BMD) of total DRA in indicated groups. mOC-AB-DBM displayed the best osteogenic mineralization at 4 weeks. n = 8 per group.

(i) DBM co-incubated with Annexin V-FITC labeled ABs were grafted in mice, and (j) the fluorescence intensity of FITC in bone regenerative area was observed using confocal microscopy.

The data in the figures represent the averages ± SD. Significant differences are indicated as * (*p* < 0.05) or ** (*p* < 0.01) paired using Student’s t test unless otherwise specified.

Table S1. Primer sequences for qPCR

| **Genes** | **Forward** | **Reverse** | **Tm (°C)** |
| --- | --- | --- | --- |
| *Runx2* | 5'-ATGCTTCATTCGCCTCACAAA-3' | 5'-GCACTCACTGACTCGGTTGG-3' | 61 |
| *Alpl* | 5'-AACCCAGACACAAGCATTCC-3' | 5'-GAGACATTTTCCCGTTCACC-3' | 60 |
| *Pecam1* | 5'-ACGCTGGTGCTCTATGCAAG-3' | 5'-TCAGTTGCTGCCCATTCATCA-3' | 62 |
| *Kdr* | 5'-GGCTAACGTGTCCTGCCAG-3' | 5'-AGTACCAACGCACAGTGATATTG-3' | 62 |
| *Ang-1* | 5'-AGTGGACACTGGACATTGCAG-3' | 5'-GCTTCCTCTTTACCATCTGTGG-3' | 60 |
| *Sp7* | 5'-CCTCTGCGGGACTCAACAAC-3' | 5'-AGCCCATTAGTGCTTGTAAAGG-3' | 60 |
| *Col1a1* | 5'-GCTCCTCTTAGGGGCCACT-3' | 5'-CCACGTCTCACCATTGGGG-3' | 61 |
| *β-actin* | 5'-TCCCTGTATGCCTCTG-3' | 5'-ATGTCACGCACGATTT-3' | 61 |
